# B-cell phenotype and IgD-CD27- memory B cells are affected by TNF-inhibitors and tocilizumab treatment in rheumatoid arthritis

**DOI:** 10.1371/journal.pone.0182927

**Published:** 2017-09-08

**Authors:** Rita A. Moura, Cláudia Quaresma, Ana R. Vieira, Maria J. Gonçalves, Joaquim Polido-Pereira, Vasco C. Romão, Nádia Martins, Helena Canhão, João E. Fonseca

**Affiliations:** 1 Rheumatology Research Unit, Instituto de Medicina Molecular, Faculdade de Medicina, Universidade de Lisboa, Lisbon, Portugal; 2 Rheumatology Department, Centro Hospitalar de Lisboa Norte, EPE, Hospital de Santa Maria, Lisbon Academic Medical Centre, Lisbon, Portugal; Monash University, AUSTRALIA

## Abstract

**Background:**

The use of TNF-inhibitors and/or the IL-6 receptor antagonist, tocilizumab, in rheumatoid arthritis (RA) have pleiotropic effects that also involve circulating B-cells. The main goal of this study was to assess the effect of TNF-inhibitors and tocilizumab on B-cell phenotype and gene expression in RA.

**Methods:**

Blood samples were collected from untreated early RA (ERA) patients, established RA patients under methotrexate treatment, established RA patients before and after treatment with TNF-inhibitors and tocilizumab, and healthy donors. B-cell subpopulations were characterized by flow cytometry and B-cell gene expression was analyzed by real-time PCR on isolated B-cells. Serum levels of BAFF, CXCL13 and sCD23 were determined by ELISA.

**Results:**

The frequency of total CD19+ B cells in circulation was similar between controls and all RA groups, irrespective of treatment, but double negative (DN) IgD-CD27- memory B cells were significantly increased in ERA and established RA when compared to controls. Treatment with TNF-inhibitors and tocilizumab restored the frequency of IgD-CD27- B-cells to normal levels, but did not affect other B cell subpopulations. TACI, CD95, CD5, HLA-DR and TLR9 expression on B-cells significantly increased after treatment with either TNF-inhibitors and/ or tocilizumab, but no significant changes were observed in BAFF-R, BCMA, CD69, CD86, CXCR5, CD23, CD38 and IgM expression on B-cells when comparing baseline with post-treatment follow-ups. Alterations in B-cell gene expression of BAFF-R, TACI, TLR9, FcγRIIB, BCL-2, BLIMP-1 and β2M were found in ERA and established RA patients, but no significant differences were observed after TNF-inhibitors and tocilizumab treatment when comparing baseline and follow-ups. Serum levels of CXCL13, sCD23 and BAFF were not significantly affected by treatment with TNF-inhibitors and tocilizumab.

**Conclusions:**

In RA patients, the use of TNF-inhibitors and/ or tocilizumab treatment affects B-cell phenotype and IgD-CD27- memory B cells in circulation, but not B-cell gene expression levels.

## Introduction

Rheumatoid arthritis (RA) is a systemic immune mediated inflammatory disease characterized by progressive joint damage. The etiology of RA is unknown, but different effector pathways and cells are involved in the cascade of events leading to the progression and persistence of the disease [1]. B cells have a critical role in the development of RA [2]. In fact, recent studies by our group have demonstrated that very early RA patients (with less than 6 weeks of disease duration) have disturbances in circulating memory B cells [3], increased levels of cytokines and B cell gene expression levels relevant for B cell maturation [4–6], which supports an active role of B cells in RA pathogenesis from early disease onset. This is reinforced by the therapeutic efficacy of rituximab, an anti-CD20 monoclonal antibody that specifically depletes B cells [7]. Other biologic treatments such as tumor necrosis factor (TNF) antagonists and the interleukin (IL)-6 receptor blocking antibody, tocilizumab, have also proven to be clinically effective in RA [8, 9] by interfering with specific cytokine dependent mechanisms. In addition, both TNF and IL-6 can have B cell regulatory effects. For instance, IL-6 plays an important role in plasma cell differentiation [10] and TNF promotes B-cell proliferation and immunoglobulin secretion [11]. Furthermore, it is known that IL-6 supports B-cell recruitment towards RA synovium [12] and TNF influences diverse pathologic processes including joint destruction and synovial hyperplasia [13]. Thus, it is plausible that some of the positive effects of TNF-inhibitors and tocilizumab on RA disease activity might be at least partially mediated by interference with B cells. In fact, preliminary reports have suggested that B cell function and humoral immune responses might be modulated by anti-TNF and tocilizumab treatments in RA [14, 15]. Therefore, the main goal of the present study was to analyze B cell phenotype and gene expression directly related with B cell activation and survival in established RA patients before and after treatment with TNF-inhibitors and tocilizumab in order to assess the effects of these agents on B cell homeostasis.

## Materials and methods

### Patients

Blood samples were collected from 13 consecutive patients with untreated polyarthritis with less than 1 year of disease duration, who fulfilled the 2010 American College of Rheumatology (ACR)/ European League Against Rheumatism (EULAR) criteria for RA and were classified as early RA (ERA). In addition, blood samples were also collected from established RA patients treated with methotrexate (MTX) (n = 20) and established RA patients before and after treatment with TNF inhibitors (Anti-TNF) (n = 10) or tocilizumab (TCZ) (n = 11). Furthermore, blood samples from 22 healthy donors were also collected and processed for comparison (Table 1). Samples were stored and managed at Biobanco-IMM, the biobank facility from Instituto de Medicina Molecular, Faculdade de Medicina, Universidade de Lisboa. All patients included in this study were attending the Rheumatology Department, Hospital de Santa Maria, Lisbon Academic Medical Centre, Portugal. The local ethics committee (Comissão de Ética do Hospital de Santa Maria, Lisbon, Portugal) approved this study and all patients and healthy donors signed an informed consent form. Patient care was conducted in accordance with standard clinical practice and the study was performed in accordance with the Declaration of Helsinki as amended in Fortaleza, Brazil (2013).

**Table 1:** Clinical characterization of patients.

	Controls	ERA	RA	Anti-TNF (n = 10)		TCZ (n = 11)	
	(n = 22)	(n = 13)	(n = 20)	Before	After	Before	After
**Age (years)**	48±8	58±14	56±13	56±15		59±15	
**Sex (% female)**	73	85	85	80		91	
**Disease duration (years)**	NA	≤ 1	8±5	14±13		10±7	
**RF (+) %**	ND	69	80	78		64	
**ACPA (+) %**	ND	62	63	38		82	
**CRP (mg/dl)**	ND	1.5±2.1	0.5±0.6	1.7±1.6	1.9±2.4	1.9±4.7	0.2±0.4#
**ESR (mm/1**^**st**^ **hour)**	ND	40±25	22±21	48±27	48±37	23±18	6±5*#&
**VAS**	NA	40±33	42±35	73±16	57±24	73±18	57±22
**DAS28**	NA	3.8±2.2	2.9±1.6	5.2±1.1**	4.0±1.5	4.9±1.7	2.9±1.2
**Swollen joints**	NA	3±4	2±4	6±3**	2±3	5±4	2±2
**Tender joints**	NA	5±5	3±5	11±9**	5±5	9±6	3±3
**Concomitant treatment:**							
**PDN**	NA	0/13	0/20	1/10		2/11	
**MTX**	NA	0/13	9/20	2/10		1/11	
**Cyclosporine**	NA	0/13	0/20	1/10		0/11	
**MTX+PDN**	NA	0/13	11/20	2/10		2/11	
**SLZ+PDN**	NA	0/13	0/20	2/10		1/11	
**LFM+PDN**	NA	0/13	0/20	1/10		0/11	
**MTX+SLZ**	NA	0/13	0/20	1/10		0/11	
**MTX+LFM**	NA	0/13	0/20	0/10		1/11	
**SLZ+HCQ+PDN**	NA	0/13	0/20	0/10		1/11	
**MTX+SLZ+HCQ+PDN**	NA	0/13	0/20	0/10		1/11	
**No treatment**	NA	13/13	0/20	0/10		2/11	

ACPA–Anti-Citrullinated Protein Antibody; CRP–C-reactive protein; DAS28 –Disease Activity Score of 28 joints; ERA–Early Rheumatoid Arthritis; ESR–Erythrocyte Sedimentation Rate; HCQ–Hydroxychloroquine; LFM–Leflunomide; MTX–Methotrexate; PDN–Prednisone; RA–Rheumatoid Arthritis; RF–Rheumatoid Factor; SLZ–Sulfasalazine; VAS–Visual Analogue Scale; NA–not applicable; ND–not determined. Values are represented as mean ± standard deviation.

* *p* < 0.05 in comparison with ERA.

** *p* < 0.05 in comparison with RA.

# *p* < 0.05 in comparison with Anti-TNF before.

& *p* < 0.05 in comparison with Anti-TNF after.

### Isolation of peripheral blood mononuclear cells

Peripheral blood mononuclear cells (PBMC) were isolated from 50 ml heparinized whole blood following density gradient centrifugation with Ficoll-Paque Plus (GE Healthcare, Sweden). Cells were washed twice in 1X phosphate buffered saline (PBS) and cellular viability was estimated with 0.4% Trypan blue (Sigma, USA).

### Flow cytometry

To analyze the frequency of B cell subpopulations in the periphery, B cells were classified using the IgD/CD27 classification system that allows the identification of four main B cell subsets (gated in CD19): naïve B cells (IgD+CD27-), pre-switch-memory (IgD+CD27+), post-switch memory (IgD-CD27+) and double-negative (DN, IgD-CD27-) B cells. A second classification system based on IgD/CD38 (gated in CD19) was also used to identify circulating transitional (IgD+CD38++) B cells and plasmablasts (IgD-CD38++). To characterize B cell phenotype, the expression of several cellular markers was analyzed, which included: BAFF-R, TACI and BCMA, the three BAFF receptors on B cells; CD69, CD86 and HLA-DR, activation markers; CXCR5, important for B cell chemotaxis; CD95, also known as Fas receptor (FasR), to analyze Fas-mediated apoptosis; IgM, a component of the B cell receptor (BCR); CD5, a marker of B cell differentiation; and toll-like receptor (TLR)-9, the main TLR expressed by B cells. Immunophenotyping of B cells was performed in PBMC samples (1×10^6^ cells/ sample) using matched combinations of anti-human murine monoclonal antibodies (mAbs) conjugated to FITC, phycoerytrin (PE), peridinin chlorophyll protein (PerCP)-Cy5.5, allophycocyanin (APC), PE-Cy7, eFlour 450 and APC-eFluor780. Combinations of anti-CD19 conjugated to PerCP-Cy5.5 or APC, anti-IgD conjugated to PE-Cy7 or FITC, anti-CD27 conjugated to eFluor450 or FITC, anti-CD38 conjugated to APC-eFluor780, anti-BAFF-R conjugated to PE, anti-TACI conjugated to APC, anti-CD86 conjugated to PE, anti-CD69 conjugated to PerCP or APC, anti-IgM conjugated to PE, anti-CD5 conjugated to APC, anti-CXCR5 conjugated to PE, anti-HLA-DR conjugated to APC, anti-CD95 conjugated to APC, anti-BCMA conjugated to PE and anti-TLR9 conjugated to APC were used. All antibodies were purchased from BD Pharmingen (USA), eBioscience (USA) and R&D Systems (United Kingdom). For cell surface stainings, PBMC were incubated with antibodies during 30 minutes, in the dark, at 4°C. For TLR9 intracellular staining, PBMC were fixed during 20 minutes at room temperature with IC Fixation Buffer (eBioscience, USA), permeabilized with 1X Permeabilization Buffer (eBioscience, USA) and stained according to eBioscience intracellular antigen staining protocol. A total of 50.000 cells/ sample gated in CD19+ B cells were acquired with LSR Fortessa (BD). Data were analyzed with FlowJo (TreeStar, Stanford University, California, USA). All samples were acquired on the same day of the staining protocol.

### B cell separation

B cells were isolated by positive MACS Separation using CD19 Microbeads and LS Columns (Miltenyi Biotec GmbH, Germany), according to the manufacturer’s instructions, using ice cold buffers and reagents to avoid cellular activation. After isolation, B cells were immediately stored at -80°C until further. Purity of isolated B cells was analyzed by flow cytometry using fluorochrome-conjugated CD20 FITC (BD Biosciences, USA) and CD3 APC (eBioscience, USA) antibodies. A total of 20.000 cells/ sample were acquired with LSR Fortessa (BD Biosciences, USA).

### RNA extraction and complementary DNA (cDNA) synthesis

Total RNA was extracted from B cells using the RNeasy Mini kit (Qiagen, Germany) according to the manufacturer’s instructions and treatment with RNase-free DNase Set (Qiagen, Germany) was performed to avoid contamination of genomic DNA. RNA concentration and purity were determined with NanoDrop ND-1000 spectrophotometer (NanoDrop Technologies, USA). Total RNA was reverse-transcribed into cDNA using DyNAmoTM cDNA Synthesis Kit for qRT-PCR (Finnzymes, Finland) with Moloney murine leukemia virus (M-MuLV) reverse transcriptase, random hexamers (300 ng/μl) and 2X RT Buffer, according to the manufacturer’s instructions, performed on Piko Thermal Cycler (Finnzymes, Finland). The cDNA samples were stored at -20°C.

### Real-time quantitative polymerase chain reaction

The expression of a group of genes directly related with B cell activation through either BAFF (BAFF-R, TACI, BCMA) or TLRs (TLR7, TLR9, TLR10), chemotaxis (CXCR5), B cell inhibition (FcγRIIB or CD32), apoptosis (BCL-2), class-switch recombination (AID), plasma cell differentiation (BLIMP-1) and cellular activation (β2M) was assessed by real-time quantitative polymerase chain reaction (qPCR) performed on Rotor-Gene 6000 (Corbett Life Science, USA) using SensiMix SYBR No-ROX Kit (Bioline, United Kingdom). The qPCR program consisted of an initial denaturation step at 95°C for 10 min, followed by 40 cycles of 95°C for 15 s, 60°C for 15 s, and 72°C for 15 s. Genes and primer sequences analyzed in this study are indicated in Table 2. Primers were designed using the National Center for Biotechnology Information (NCBI)/ Primer-BLAST. The 18S ribosomal RNA (18S rRNA) was used as endogenous control in relative quantification using the standard curve method. All data were analyzed with Rotor-Gene 6000 Series Software.

**Table 2:** Genes and sequences of primers used in quantitative real time PCR.

Gene	Name	Primer sequences
**18S rRNA**	18S ribossomal RNA	Fw: 5’-GGAGTATGGTTGCAAAGCTGA-3’ Rv: 5’-ATCTGTCAATCCTGTCCGTGT-3’
**BAFF-R**	BAFF receptor	Fw: 5’-CTGGTCCTGGTGGGTCTG-3’ Rv: 5’-ACCTTGTCCAGGGGCTCT-3’
**TACI**	Transmembrane activator and calcium modulator cyclophilin ligand interactor	Fw: 5’-AGGCTCAGAAGCAAGTCCAG-3’ Rv: 5’-CCAGGAAGCAGCAGAGGA-3’
**BCMA**	B cell maturation antigen	Fw: 5’-AGGACGAGTTTAAAAACACAGGA-3’ Rv: 5’-TCACAGGTGCATTCTTCCAC-3’
**CXCR5**	C-X-C chemokine receptor 5	Fw: 5’-GGAGCCTCTCAACATAAGACAGT-3’ Rv: 5’-ATTTTCCACCAGGGAGGTGTC-3’
**AID**	Activation-induced cytidine deaminase	Fw: 5’-GGACTTTGGTTATCTTCGCAAT-3’ Rv: 5’-GTCGGGCACAGTCGTAGC-3’
**BLIMP-1**	B lymphocyte-induced maturation protein	Fw: 5’-ACGTGTGGGTACGACCTTG-3’ Rv: 5’-CTGCCAATCCCTGAAACCT-3’
**β2M**	Beta 2-microglobulin	Fw: 5’-CTATCCAGCGTACGCCAAAGATTC-3’ Rv: 5’-CTTGCTGAAAGACAAGTCTGAATG-3’
**BCL-2**	B-cell lymphoma 2	Fw: 5’-TTGACAGAGGATCATGCTGTACTT-3’ Rv: 5’-ATCTTTATTTCATGAGGCACGTT-3’
**FcγRIIB (CD32)**	Fc-gamma receptor IIB	Fw: 5’-GTGCTATTCCTGGCTCCTGTT-3’ Rv:5’-CGTGTGGGTGGGAATGAGAT-3’
**TLR7**	Toll-like receptor 7	Fw: 5’-CACCTCTCATGCTCTGCTCTC-3’ Rv: 5’-TCTAGCCCCAAGGAGTTTGGA-3´’
**TLR9**	Toll-like receptor 9	Fw: 5’-GGACCTCGAGTGTGAAGCAT-3’ Rv: 5’-TGGAGCTCACAGGGTAGGAA-3’
**TLR10**	Toll-like receptor 10	Fw: 5’-TCTGCTGAGAGAGTGCAAGC-3’ Rv: 5’-ACATGTTGGAGCAGTTGGTCA-3’

### Enzyme-linked immunosorbent assay (ELISA)

B cell activating factor (BAFF) (Bender MedSystems, Austria), B-lymphocyte chemoattractant (BLC) also known as C-X-C motif chemokine 13 (CXCL13) and the soluble form of CD23 (sCD23) (R&D systems, United Kingdom) were quantified in serum samples from all groups by ELISA according to the manufacturer’s instructions. Samples were analyzed using plate reader Infinite M200 (Tecan, Switzerland).

### Statistical analysis

Statistical differences were determined with GraphPad Prism (GraphPad, San Diego, USA). For populations that did not follow a Gaussian distribution, non-parametric tests were used. The Mann-Whitney test was used for comparisons between 2 independent groups. For comparisons between 3 or more groups, the Kruskal-Wallis and Dunn’s multiple comparison tests were used. The Wilcoxon matched pairs test was used for comparisons between 2 paired groups. Correlation analyses were performed using Spearman’s test. Differences were considered statistically significant for p < 0.05.

## Results

### Clinical characterization of patients

A group of untreated polyarthritis patients (n = 13), with less than 1 year of disease duration, fulfilling the 2010 ACR/ EULAR criteria for RA, was included in this study and was classified as early RA (ERA). ERA patients had a disease activity score of 28 joints (DAS28) of 3.8±2.2. A group of established RA patients, under MTX treatment (n = 20), with a DAS28 of 2.9±1.6, was also included. Moreover, established RA patients that had either initiated treatment with TNF-inhibitors (n = 10) or tocilizumab (n = 11) were evaluated at baseline and after an average follow-up of 8 months of treatment (minimum 3 months). At baseline, RA patients treated with TNF-inhibitors had a DAS28 of 5.2±1.1 and, at post-treatment, 4.0±1.5. Fifty percent of the patients had initiated etanercept, 40% golimumab and 10% adalimumab. RA patients treated with tocilizumab had at baseline a DAS28 of 4.9±1.7 and, at post-treatment, 2.9±1.2. All patients were concomitantly being treated with synthetic disease modifying anti-rheumatic drugs (DMARDs). Most patients were rheumatoid factor (RF) and anti-citrullinated protein antibody (ACPA) seropositive in all studied groups. In addition, a group of age and sex-matched healthy donors (n = 22) was included. The clinical information from all patients and data from healthy controls included in this study are indicated in Table 1.

### Early and established RA patients have alterations in memory B cell subpopulations in peripheral blood when compared to healthy individuals

**Fig 1:**
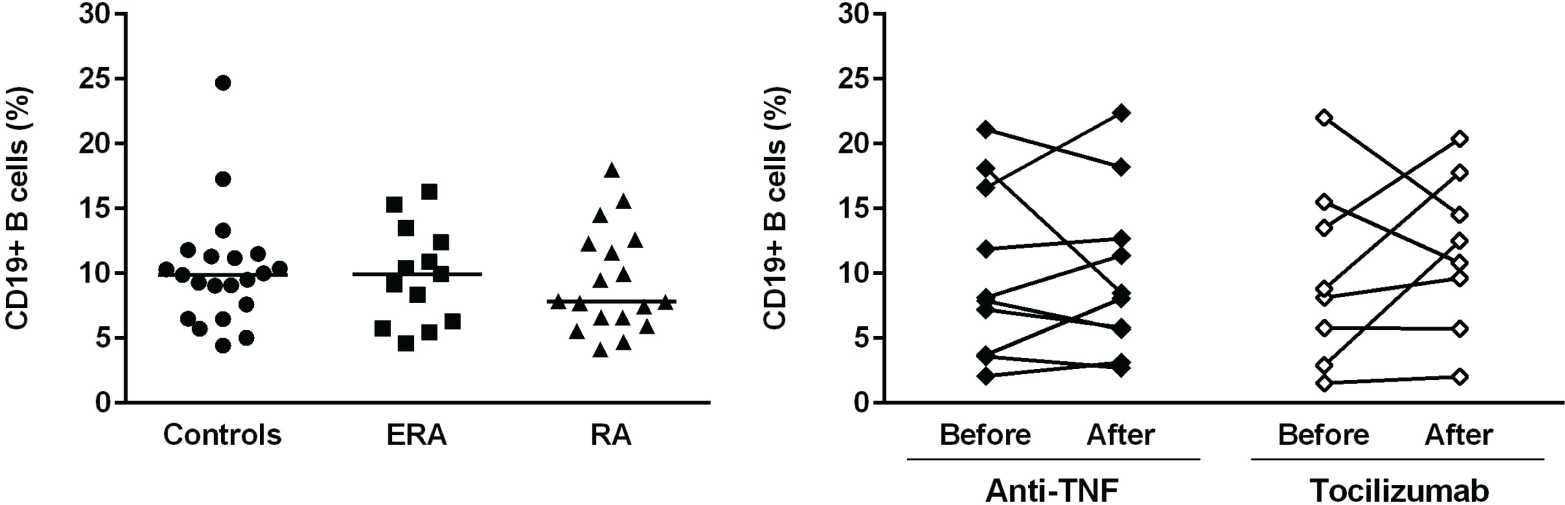
Peripheral blood CD19+ B cell levels are not affected by treatment with TNF-inhibitors and tocilizumab in rheumatoid arthritis. The frequency of total CD19+ B cells was determined by flow cytometry in early RA (ERA) and established RA patients under methotrexate treatment. In addition, the effect of TNF-inhibitors and tocilizumab treatment on circulating B cells was also assessed in established RA patients at baseline and after an average of 8 months of treatment. A group of healthy individuals was also included as controls. Lines represent median values. Differences were considered statistically significant for *p*<0.05. Non-parametric Mann-Whitney test was used for comparisons between 2 independent groups. For paired samples (before and after treatment), the Wilcoxon signed-rank test was used.

**Fig 2:**
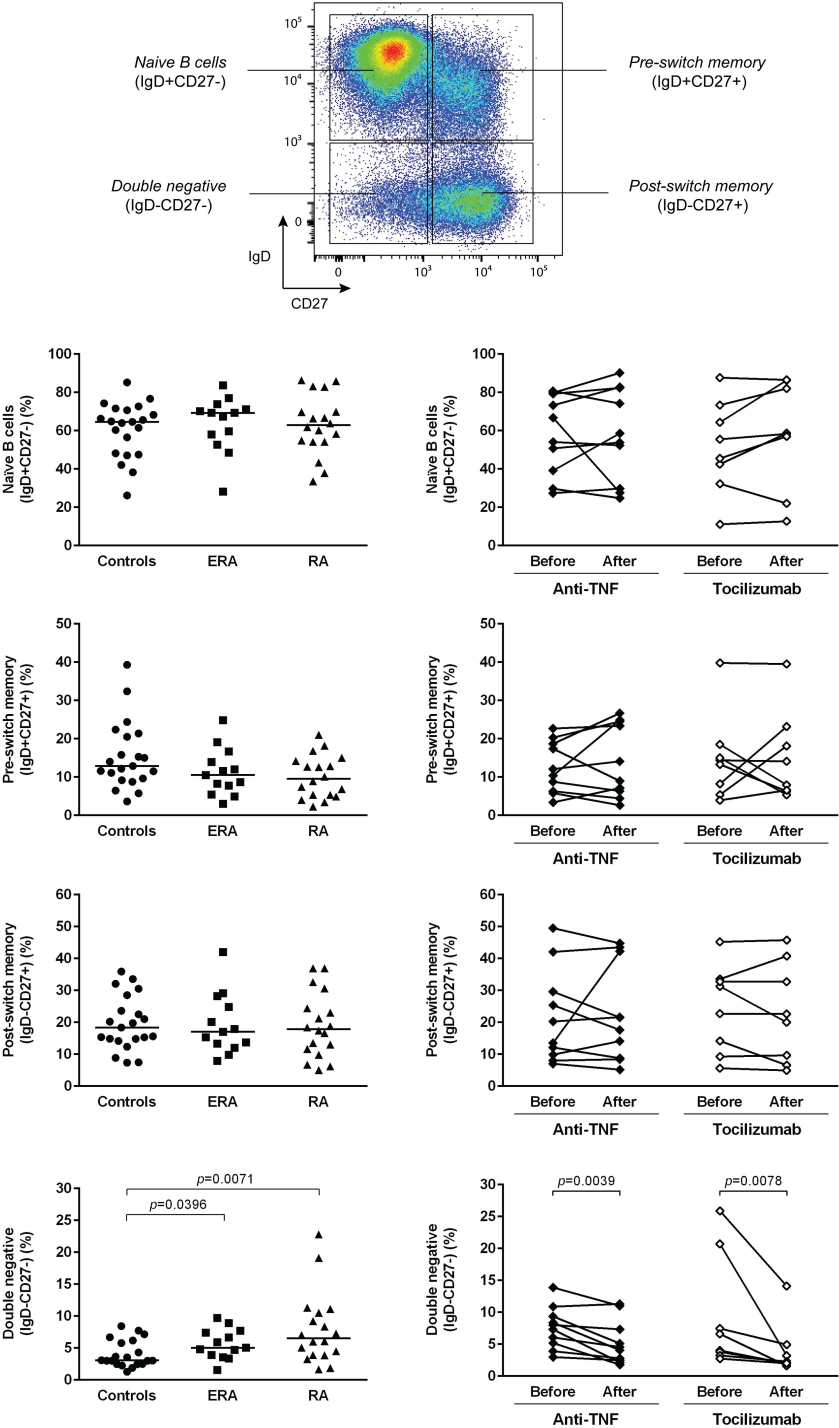
In rheumatoid arthritis, treatment with TNF-inhibitors and tocilizumab restores the frequency of IgD-CD27- memory B cells to normal levels. The frequency of B cell subpopulations based on IgD/ CD27 classification was determined by flow cytometry in early RA (ERA) and established RA patients under methotrexate treatment. In addition, the effect of TNF-inhibitors and tocilizumab treatment on circulating B cell subsets was also assessed in established RA patients at baseline and after an average of 8 months of treatment. A group of healthy individuals was also included as controls. Lines represent median values. Gating strategy for B cell subpopulations (defined in CD19+ B cells) based on IgD/ CD27 classification is shown. Differences were considered statistically significant for *p*<0.05. Non-parametric Mann-Whitney test was used for comparisons between 2 independent groups. For paired samples (before and after treatment), the Wilcoxon signed-rank test was used.

**Fig 3:**
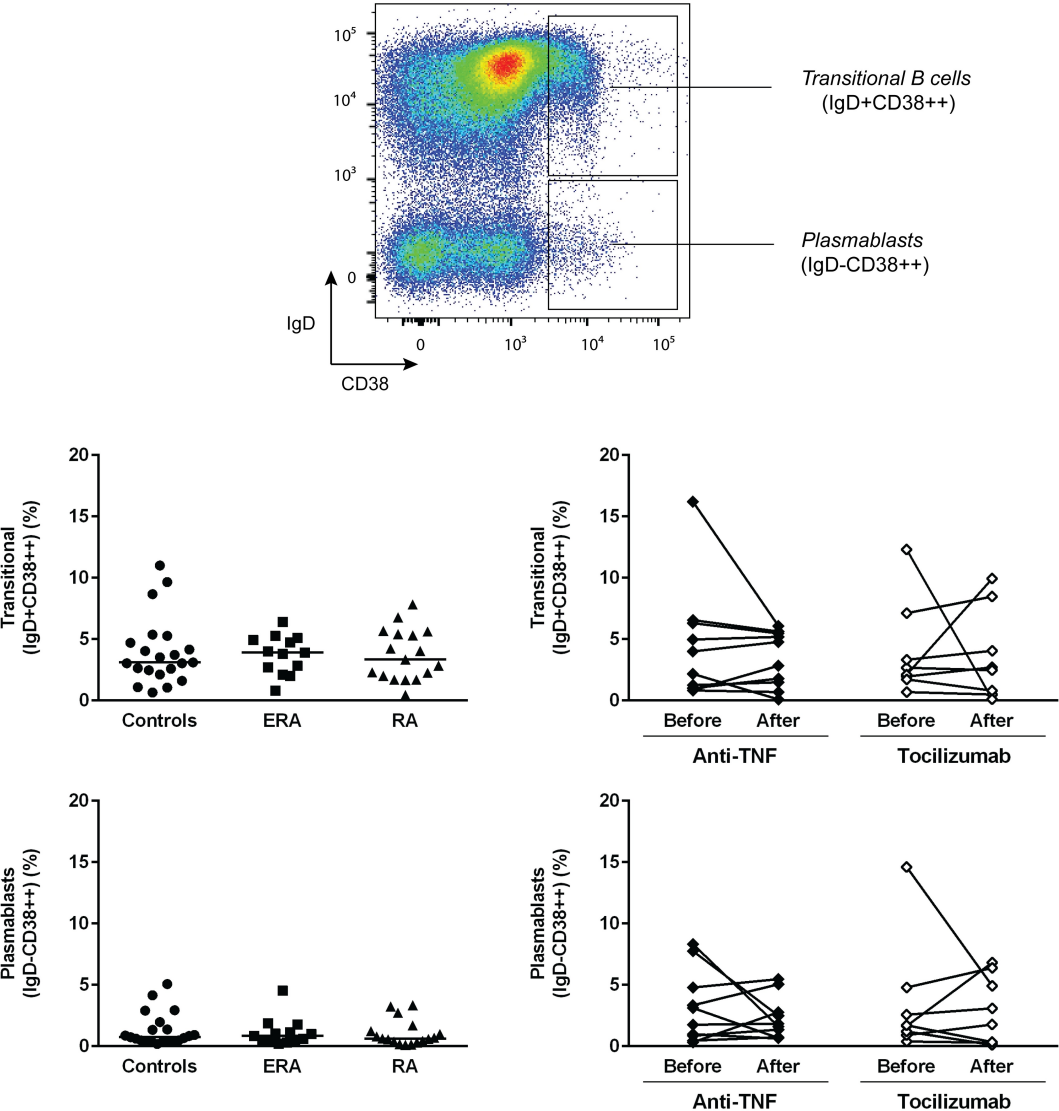
Transitional B cells and plasmablasts in circulation are not affected by treatment with TNF-inhibitors and tocilizumab in rheumatoid arthritis. The frequency of peripheral blood transitional B cells and plasmablasts based on IgD/ CD38 classification was determined by flow cytometry in early RA (ERA) and established RA patients under methotrexate treatment. In addition, the effect of TNF-inhibitors and tocilizumab treatment on these circulating B cell subsets was also assessed in established RA patients at baseline and after an average of 8 months of treatment. A group of healthy individuals was also included as controls. Lines represent median values. Gating strategy for B cell subpopulations (defined in CD19+ B cells) based on IgD/ CD38 classification is shown. Differences were considered statistically significant for *p*<0.05. Non-parametric Mann-Whitney test was used for comparisons between 2 independent groups. For paired samples (before and after treatment), the Wilcoxon signed-rank test was used.

The analysis of the frequency of total CD19+ B cells has revealed no statistically significant differences between ERA, established RA patients and healthy controls. Furthermore, no effect of TNF-inhibitors and tocilizumab treatment was observed when comparing baseline and follow-ups (Fig 1). ERA and established RA patients had similar circulating levels of transitional (IgD+CD38++), naïve (IgD+CD27-), pre-switch memory B cells (IgD+CD27+), post-switch memory B cells (IgD-CD27+) and plasmablasts (IgD-CD38+++) in comparison with controls and no significant differences were observed between the two patient groups (Figs 2 and 3). In addition, both ERA and established RA patients had significantly higher frequencies of DN (IgD-CD27-) B cells when compared to controls (Fig 2). Treatment with TNF-inhibitors and tocilizumab did not significantly affect B cell subpopulations in circulation, except DN (IgD-CD27-) B cells, whose frequencies were significantly lower when comparing baseline and follow-ups (Figs 2 and 3). In fact, both TNF-inhibitors and tocilizumab treatment restored the frequency of DN (IgD-CD27-) B cells to normal values when compared to healthy controls [median (range) of DN (IgD-CD27-) B cells 4.3 (1.8–11.3) % after TNF-inhibitors; 2.2 (1.6–14.1) % after tocilizumab; 3.1 (1.3–8.4) % controls, respectively] (Fig 2). The majority of DN (IgD-CD27-) B cells was class-switched, since an average of 5% of these cells were IgM positive in all groups analyzed (S1 Fig). Furthermore, IgM expression by IgD-CD27- B cells was not significantly affected by treatment with TNF-inhibitors, but decreased after tocilizumab (S1 Fig). Moreover, no significant correlations were found between the frequencies of all B cell subpopulations with age, disease activity score (DAS28), or with clinical parameters, namely erythrocyte sedimentation rate (ESR), C-reactive protein (CRP), swollen and tender joint counts, or disease duration in all studied groups (data not shown).

### Treatment with TNF-inhibitors and tocilizumab affects the expression of B cell markers in RA patients

**Fig 4:**
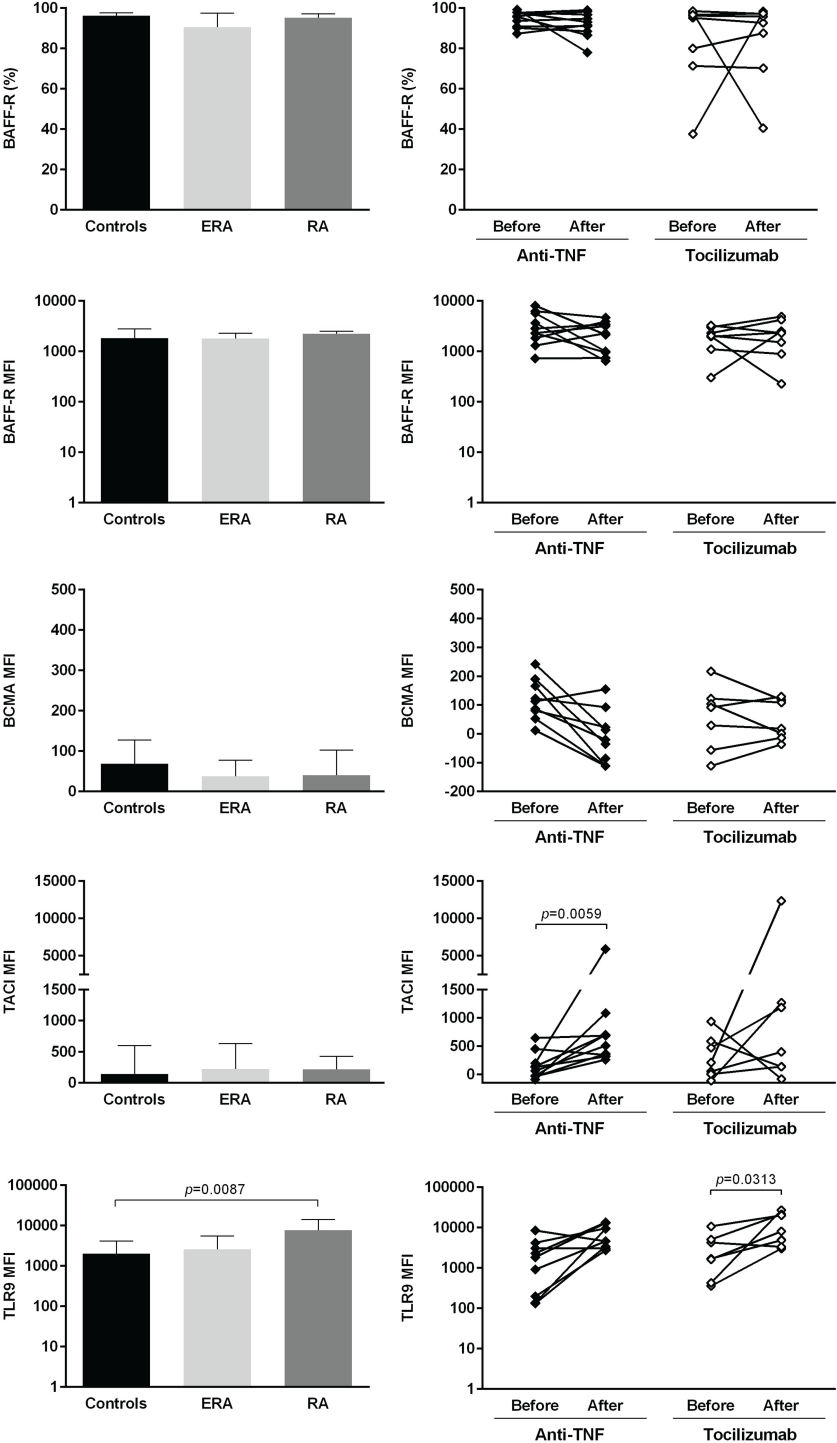
TACI, but not BAFF-R or BCMA, and TLR9 B-cell expression increase after treatment with TNF-inhibitors and tocilizumab in rheumatoid arthritis. The expression of B-cell activating factor receptors (BAFF-R, BCMA, TACI) and toll-like receptor 9 (TLR9) was analyzed on B-cells (frequency and median fluorescence intensity, MFI) to characterize B-cell phenotype in circulation in early RA (ERA) and established RA patients under methotrexate treatment. The effect of TNF-inhibitors and tocilizumab treatment on B-cell markers expression was also assessed in established RA patients at baseline and after an average of 8 months of treatment. Bars represent median values with interquartile range. Differences were considered statistically significant for *p*<0.05. Non-parametric Mann-Whitney test was used for comparisons between 2 independent groups. For paired samples (before and after treatment), the Wilcoxon signed-rank test was used.

**Fig 5:**
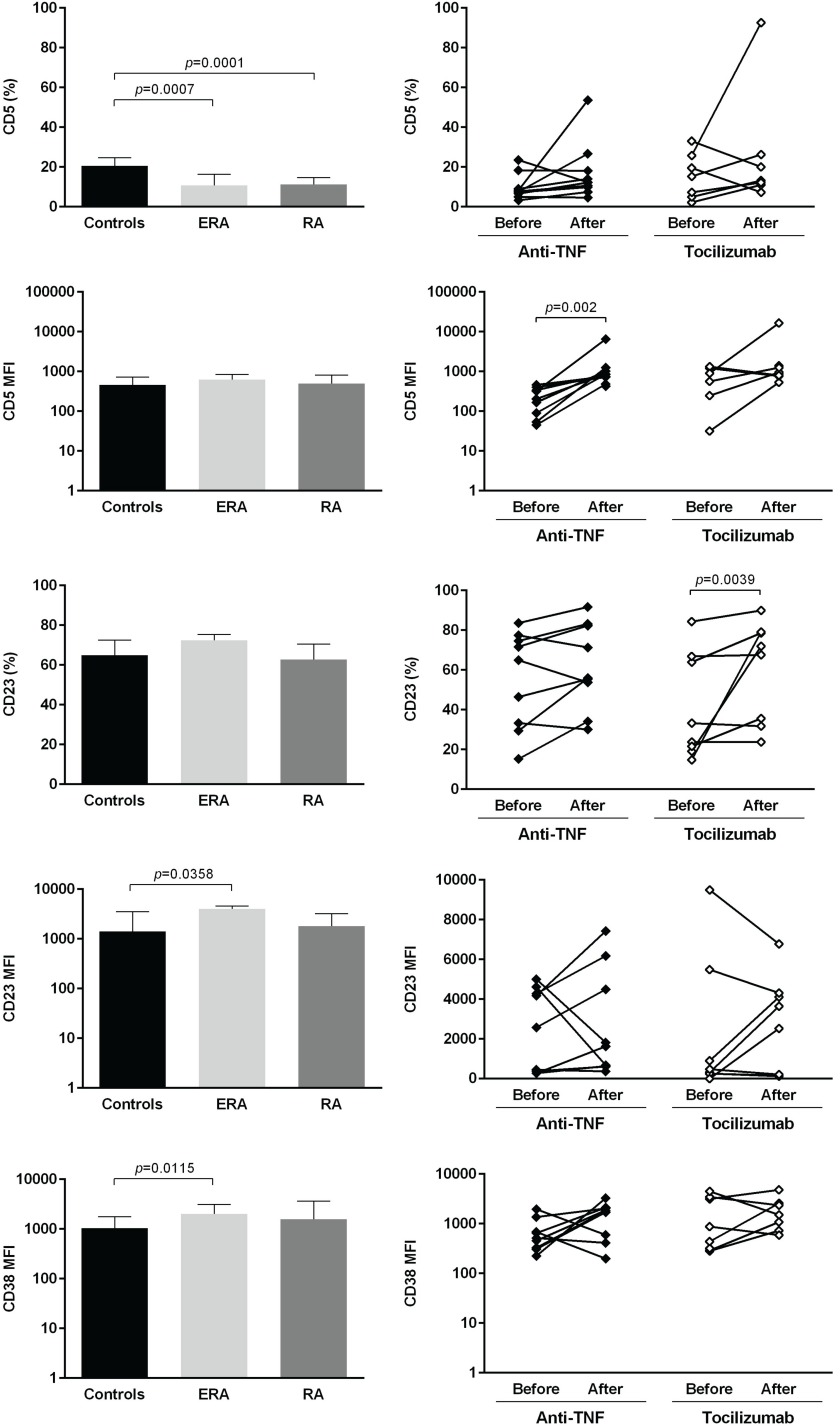
In rheumatoid arthritis, treatment with TNF-inhibitors and tocilizumab does not affect the frequency of CD5+ B cells in circulation, but CD5 MFI increases. The expression of B-cell differentiation (CD5), maturation (CD23) and calcium signaling (CD38) cell markers was analyzed on B-cells (frequency and median fluorescence intensity, MFI) to characterize B-cell phenotype in circulation in early RA (ERA) and established RA patients under methotrexate treatment. The effect of TNF-inhibitors and tocilizumab treatment on B-cell markers expression was also assessed in established RA patients at baseline and after an average of 8 months of treatment. Bars represent median values with interquartile range. Differences were considered statistically significant for *p*<0.05. Non-parametric Mann-Whitney test was used for comparisons between 2 independent groups. For paired samples (before and after treatment), the Wilcoxon signed-rank test was used.

**Fig 6:**
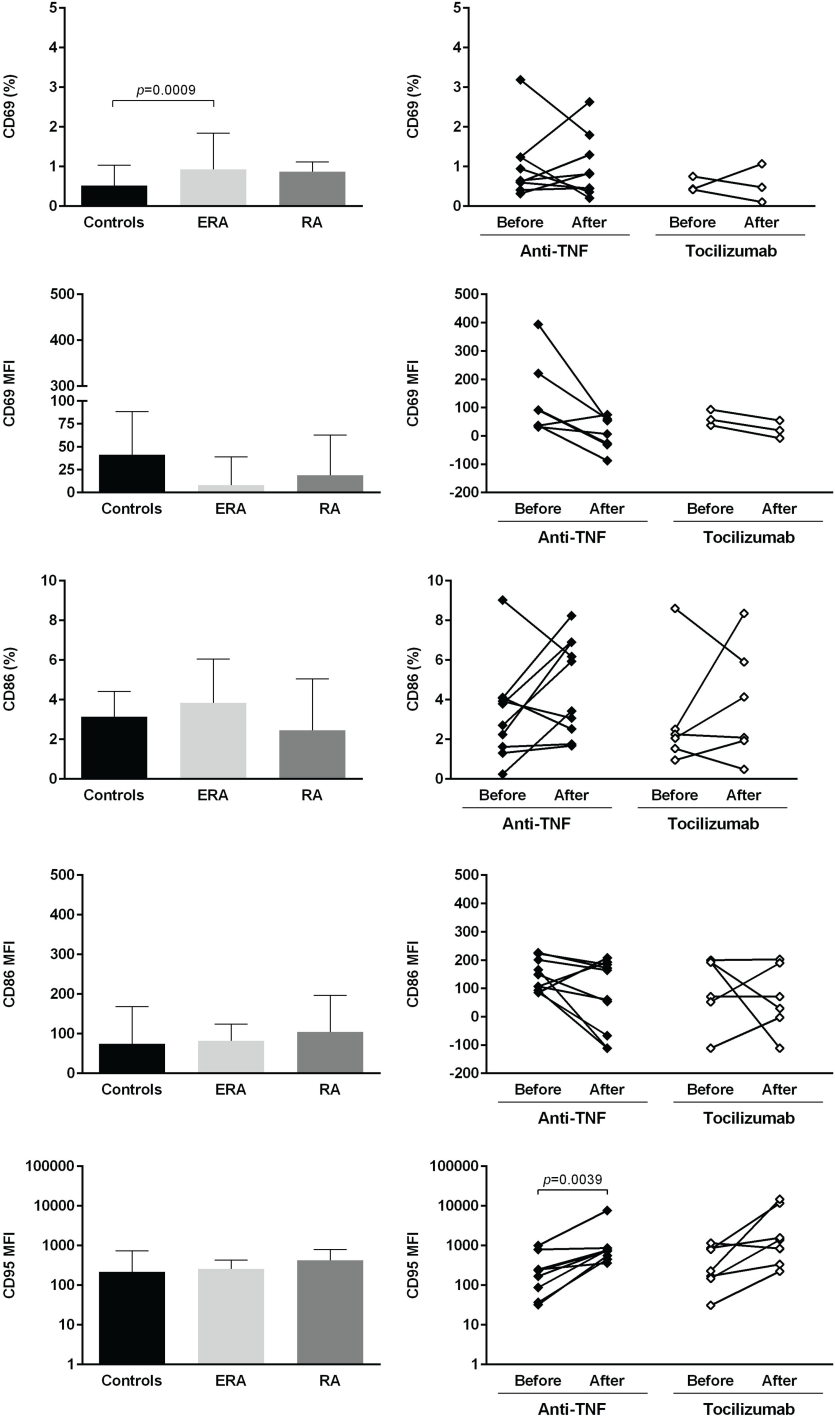
CD95, but not CD69 or CD86 B-cell expression increase after treatment with TNF-inhibitors and tocilizumab in rheumatoid arthritis. The expression of activation (CD69, CD86) and apoptosis (CD95) cell markers was analyzed on B-cells (frequency and median fluorescence intensity, MFI) to characterize B-cell phenotype in circulation in early RA (ERA) and established RA patients under methotrexate treatment. The effect of TNF-inhibitors and tocilizumab treatment on B-cell markers expression was also assessed in established RA patients at baseline and after an average of 8 months of treatment. Bars represent median values with interquartile range. Differences were considered statistically significant for *p*<0.05. Non-parametric Mann-Whitney test was used for comparisons between 2 independent groups. For paired samples (before and after treatment), the Wilcoxon signed-rank test was used.

**Fig 7:**
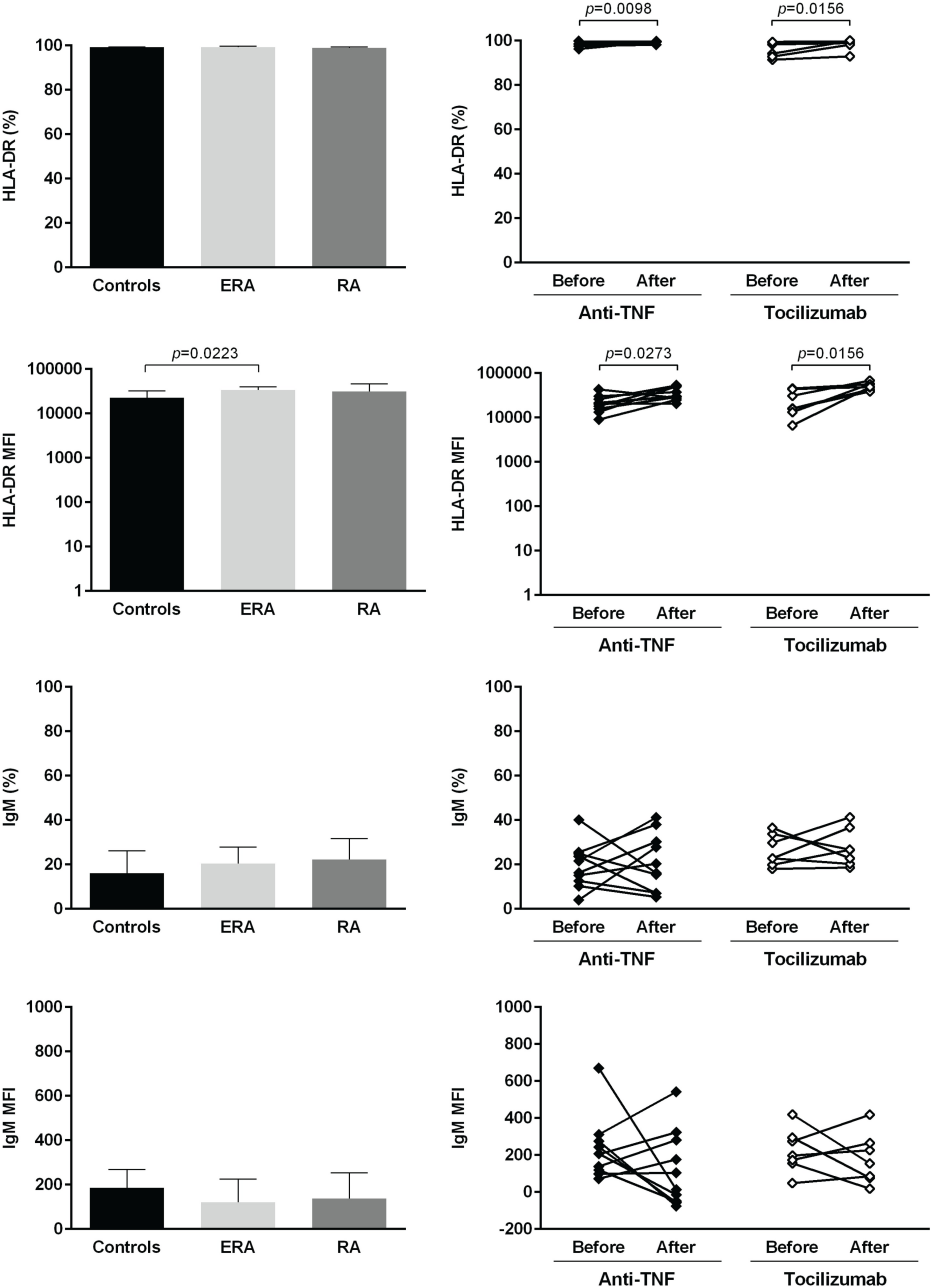
The frequency of HLA-DR+ B cells and HLA-DR MFI increase after treatment with TNF-inhibitors and tocilizumab in rheumatoid arthritis. The expression of cell markers related to antigen presentation (HLA-DR) and B-cell receptor (IgM) was analyzed on B-cells (frequency and median fluorescence intensity, MFI) to characterize B-cell phenotype in circulation in early RA (ERA) and established RA patients under methotrexate treatment. The effect of TNF-inhibitors and tocilizumab treatment on B-cell markers expression was also assessed in established RA patients at baseline and after an average of 8 months of treatment. Bars represent median values with interquartile range. Differences were considered statistically significant for *p*<0.05. Non-parametric Mann-Whitney test was used for comparisons between 2 independent groups. For paired samples (before and after treatment), the Wilcoxon signed-rank test was used.

**Fig 8:**
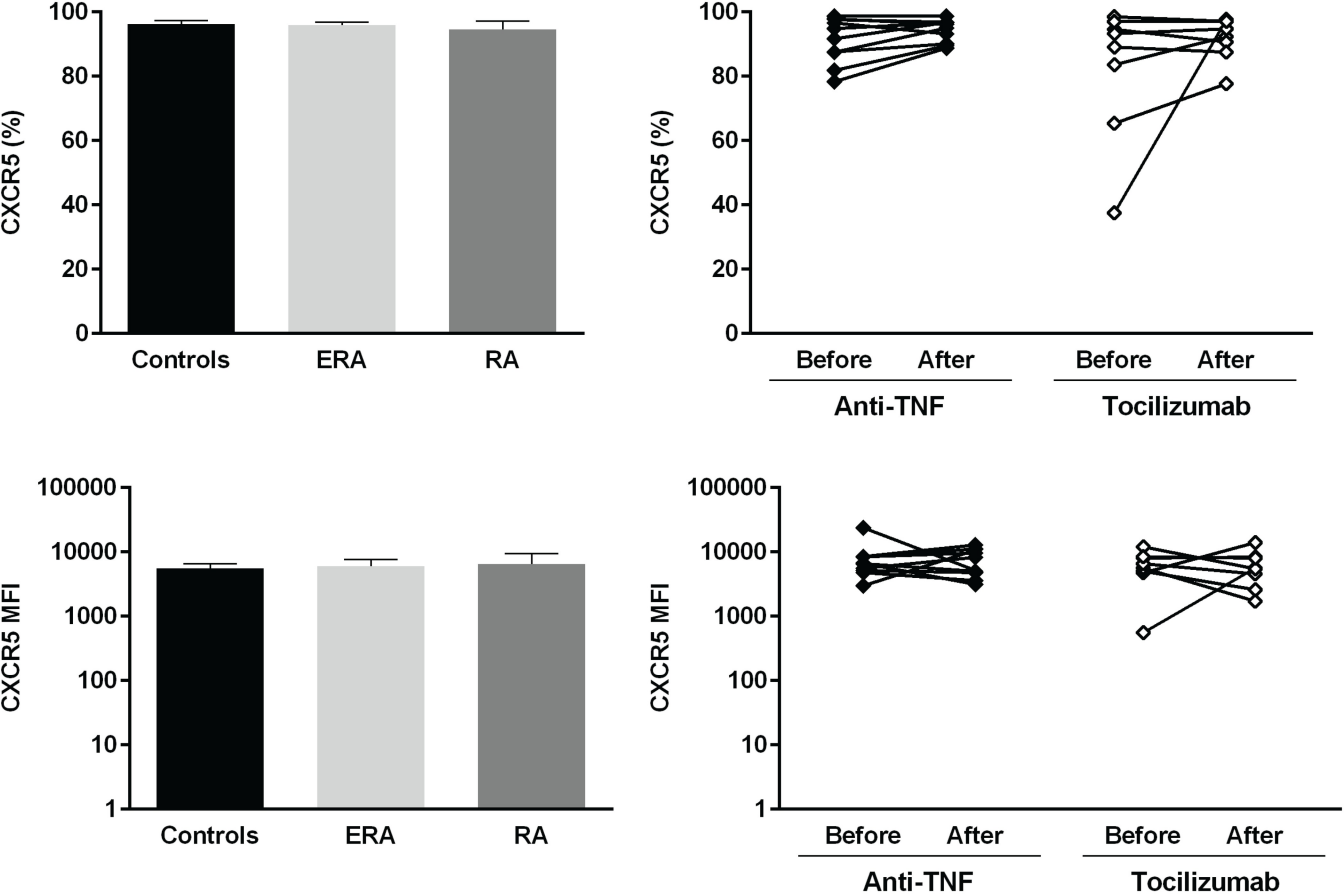
Treatment with TNF-inhibitors and tocilizumab does not affect the circulating levels of CXCR5+ B cells or CXCR5 MFI in rheumatoid arthritis. The expression of chemokine receptor CXCR5 was analyzed on B-cells (frequency and median fluorescence intensity, MFI) to characterize B-cell phenotype in circulation in early RA (ERA) and established RA patients under methotrexate treatment. The effect of TNF-inhibitors and tocilizumab treatment on B-cell markers expression was also assessed in established RA patients at baseline and after an average of 8 months of treatment. Bars represent median values with interquartile range. Differences were considered statistically significant for *p*<0.05. Non-parametric Mann-Whitney test was used for comparisons between 2 independent groups. For paired samples (before and after treatment), the Wilcoxon signed-rank test was used.

The expression of several cellular markers (frequency, % and median fluorescence intensity, MFI) was analyzed to characterize B cell phenotype in circulation (Figs 4–8). It was observed that BAFF-R, TACI and BCMA expression were similar between all studied groups (Fig 4). Treatment with TNF inhibitors and tocilizumab did not significantly affect the expression of these markers when comparing baseline and follow-ups, except TACI, whose MFI values significantly increased after anti-TNF therapy (Fig 4). The expression of TLR9 was similar between ERA patients and healthy donors, but it was significantly increased in established RA when compared to controls (Fig 4). Furthermore, TLR9 MFI values significantly increased in RA patients after tocilizumab, but not after treatment with TNF-inhibitors (Fig 4). ERA and established RA patients had significantly lower levels of CD5+ B cells in circulation when compared to controls, although no significant differences were detected in CD5 MFI values (Fig 5). No effect of treatment with TNF-inhibitors and tocilizumab was observed in the frequency of CD5+ B cells, but CD5 MFI significantly increased after TNF-inhibitors, but not tocilizumab treatment, when comparing baseline and follow-ups (Fig 5). The frequency of CD23+ B cells in circulation was similar between ERA, established RA and controls. However, CD23 MFI values were significantly increased in ERA, but not established RA patients, when compared to controls (Fig 5). Although no effect of treatment with TNF-inhibitors and tocilizumab was observed in CD23 MFI values, the frequency of CD23+ B cells significantly increased after tocilizumab, but not TNF-inhibitors treatment, when comparing baseline and follow-ups (Fig 5). In addition, ERA, but not established RA patients, had significantly increased CD38 MFI levels when compared to controls and no effect of treatment with TNF-inhibitors and tocilizumab was detected (Fig 5). Moreover, ERA, but not established RA patients, had significantly increased levels of CD69+ B cells in circulation when compared to controls, but no significant differences were detected in CD69 MFI values (Fig 6). Also, no effect of TNF-inhibitors and tocilizumab treatment was observed when comparing baseline and follow-ups (Fig 6). CD86 expression (both frequency and MFI) was similar between all studied groups and no effect of treatment with TNF-inhibitors and tocilizumab was detected (Fig 6). Although no significant differences were observed in CD95 MFI values between ERA, established RA and controls, CD95 expression significantly increased in RA patients after TNF-inhibitors, but not after tocilizumab treatment (Fig 6). The frequency of HLA-DR+ B cells was similar between all patient groups when compared to controls. Nevertheless, ERA, but not established RA patients, had significantly increased HLA-DR MFI values when compared to healthy individuals (Fig 7). Furthermore, HLA-DR expression (both frequency and MFI) significantly increased in RA patients after treatment with TNF-inhibitors and tocilizumab, when comparing baseline and follow-ups (Fig 7). Additionally, IgM and CXCR5 expression (both frequency and MFI) were similar between all studied groups and no effect of treatment with TNF-inhibitors and tocilizumab was detected (Figs 7 and 8, respectively). No significant correlations were observed between the frequency or MFI values of any of the B cell markers studied with age, DAS28, ESR, CRP, swollen and tender joint counts, or disease duration in all groups analyzed (data not shown).

### Changes in B cell gene expression occur in RA patients, but no effect of treatment with TNF-inhibitors and tocilizumab is observed

**Fig 9:**
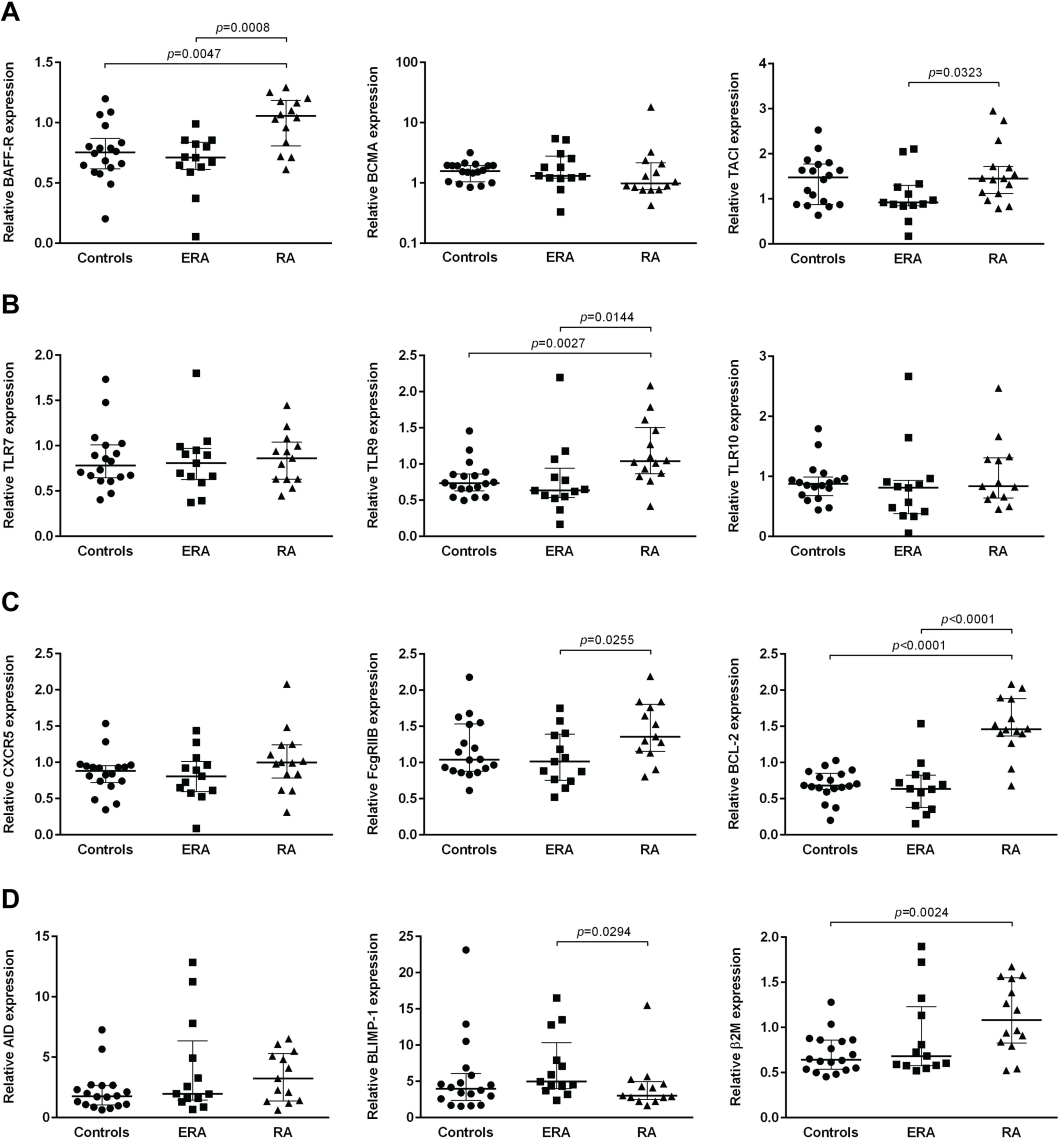
Changes in B-cell gene expression levels occur in rheumatoid arthritis, but no effect of TNF-inhibitors and tocilizumab treatment is observed. The expression of a group of genes related with B-cell activation through B-cell activating factor receptors (BAFF-R, BCMA, TACI) **(A)**; toll-like receptors (TLR7, TLR9, TLR10) **(B)**; chemotaxis (CXCR5), inhibition (FcgRIIB), apoptosis (BCL-2) **(C)**; class-switching (AID), plasma cell differentiation (BLIMP-1) and cellular activation (β2M) **(D)** was analyzed on isolated B-cells by real time PCR in early RA (ERA) and established RA. The effect of TNF-inhibitors and tocilizumab treatment on B-cell gene expression was assessed in established RA patients at baseline and after an average of 8 months of treatment. Lines represent median values with interquartile range. Differences were considered statistically significant for *p*<0.05 (non-parametric Mann-Whitney test).

The expression of a group of genes directly related with B cell activation and survival was analyzed in all studied groups (Fig 9). It was observed that BAFF-R gene expression was significantly higher in established RA patients in comparison with both ERA and controls (Fig 9A). TACI gene expression was significantly higher in established RA when compared to ERA, but no significant differences were found in comparison with controls. No significant differences were observed in BCMA gene expression between ERA, established RA and controls (Fig 9A). TLR9 gene expression levels were significantly higher in established RA when compared not only to ERA patients, but also to controls. However, no significant differences were detected in TLR7 and TLR10 B cell gene expression levels between ERA patients, established RA patients and controls (Fig 9B). FcγRIIB B cell gene expression levels were significantly higher in established RA when compared to ERA patients, but no significant differences were detected when compared to controls (Fig 9C). BCL-2 gene expression levels were significantly higher in established RA when compared to both ERA patients and controls, and no significant differences were detected in CXCR5 B cell gene expression levels between all groups (Fig 9C). Moreover, although ERA patients had similar BLIMP-1 B cell gene expression levels when compared to controls, the expression of this gene was significantly higher in ERA when compared to established RA patients (Fig 9D). β2M B cell gene expression was significantly higher in established RA patients when compared to controls, but no significant differences were detected in ERA patients in comparison, not only with controls, but also with established RA patients (Fig 9D). Furthermore, AID B cell gene expression levels were similar between all groups analyzed (Fig 9D). Of note, treatment with TNF-inhibitors and tocilizumab did not significantly affect B cell gene expression levels of any of the analyzed genes, when comparing baseline and follow-ups (data not shown). In addition, no significant correlations were found between B cell gene expression levels with age, DAS28, ESR, CRP, swollen and tender joint counts, or disease duration in all groups analyzed (data not shown).

### CXCL13 and sCD23 serum levels are increased since early RA onset and do not change after treatment with TNF inhibitors and tocilizumab

**Fig 10:**
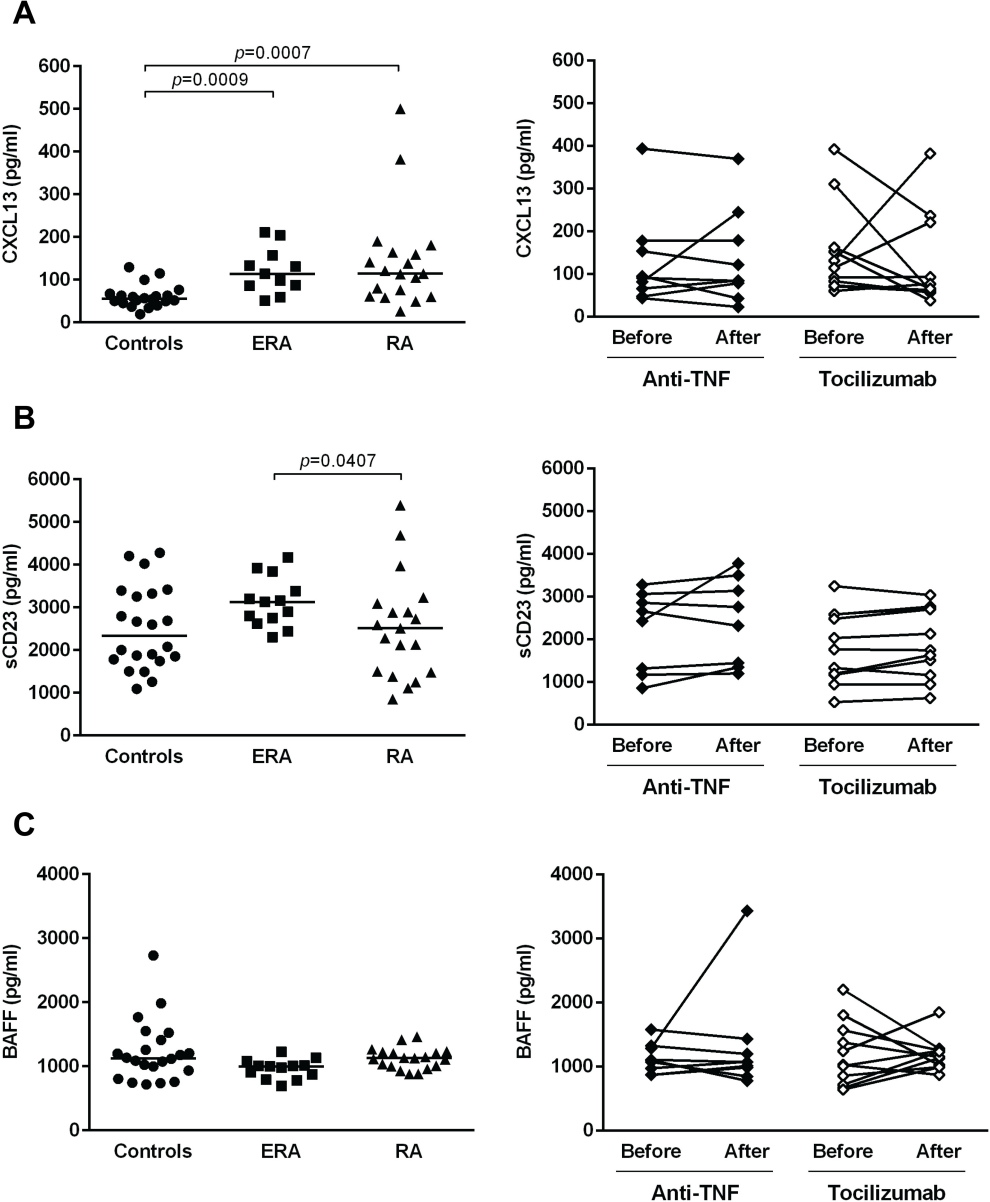
CXCL13 and sCD23 serum levels are increased since early rheumatoid arthritis onset and do not change after treatment. The serum levels of C-X-C motif chemokine 13 (CXCL13) **(A)**, soluble CD23 (sCD23) **(B)** and B cell activating factor (BAFF) **(C)** were quantified in early RA (ERA) and established RA patients under methotrexate treatment by ELISA. The effect of TNF-inhibitors and tocilizumab treatment in the production of these serological markers was also assessed in established RA patients at baseline and after an average of 8 months of treatment. Lines represent median values. Differences were considered statistically significant for *p*<0.05. Non-parametric Mann-Whitney test was used for comparisons between 2 independent groups. For paired samples (before and after treatment), the Wilcoxon signed-rank test was used.

The serum levels of CXCL13, a chemokine important for B cell chemotaxis and a ligand for CXCR5; sCD23, a marker of B cell maturation, and BAFF, a cytokine important for B cell survival, were also quantified in this study to analyze the effect of TNF-inhibitors and tocilizumab in the production of serological markers relevant for B cell activation (Fig 10). It was observed that CXCL13 serum levels were significantly higher in ERA and established RA patients when compared to healthy controls, but no significant differences were detected between both patient groups (Fig 10A). Moreover, treatment with TNF-inhibitors and tocilizumab did not affect CXCL13 circulating levels in RA patients when comparing baseline and follow-ups (Fig 10A). In addition, it was found that ERA patients had significantly higher sCD23 serum levels when compared to established RA patients, but no significant differences were observed in comparison with controls (Fig 10B). Furthermore, treatment with TNF-inhibitors and tocilizumab did not affect sCD23 circulating levels in RA patients when comparing baseline and follow-ups (Fig 10B). BAFF serum levels were similar between all groups analyzed and no effect of TNF-inhibitors and tocilizumab treatments was observed (Fig 10C). Of note, no significant correlations were detected between CXCL13, sCD23 and BAFF serum levels with B cell subpopulations in circulation, B cell markers expression, B cell gene expression, or with age, DAS28, ESR, CRP, swollen and tender joint counts, or disease duration in all groups analyzed (data not shown).

### Biologic therapy affects blood memory B cells and B cell phenotype of rheumatoid arthritis patients irrespective of response to treatment

**Fig 11:**
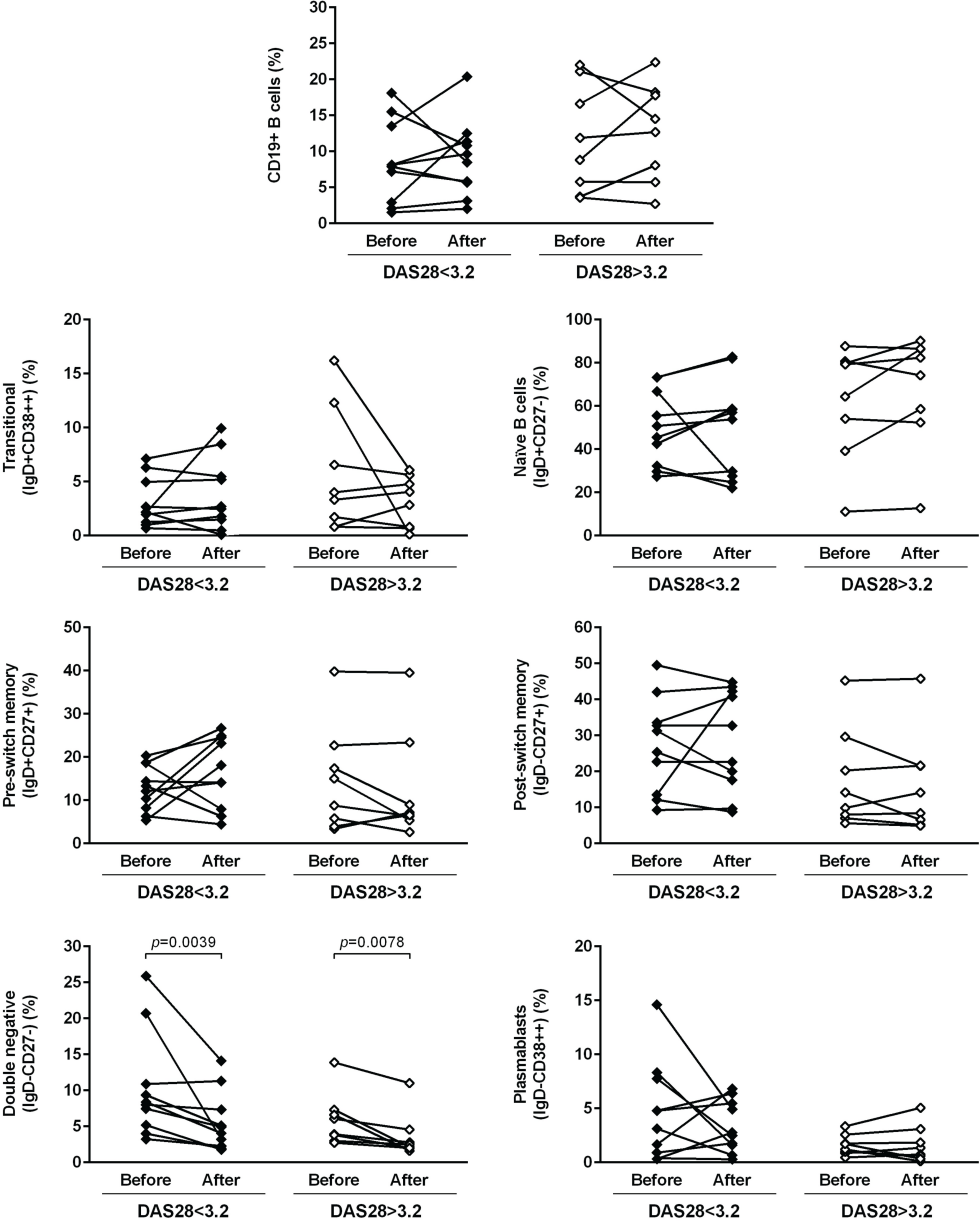
Biologic therapy affects peripheral blood memory B cells in rheumatoid arthritis patients irrespective of response to treatment. The frequency of peripheral blood B cell subpopulations was analyzed by flow cytometry in established RA patients before and after an average of 8 months of biologic therapy with TNF-inhibitors and/ or tocilizumab, based on whether the patients presented a low to moderate disease activity (DAS28<3.2) or high disease activity (DAS28>3.2) after treatment. Differences were considered statistically significant for *p*<0.05 (Wilcoxon signed-rank test).

**Fig 12:**
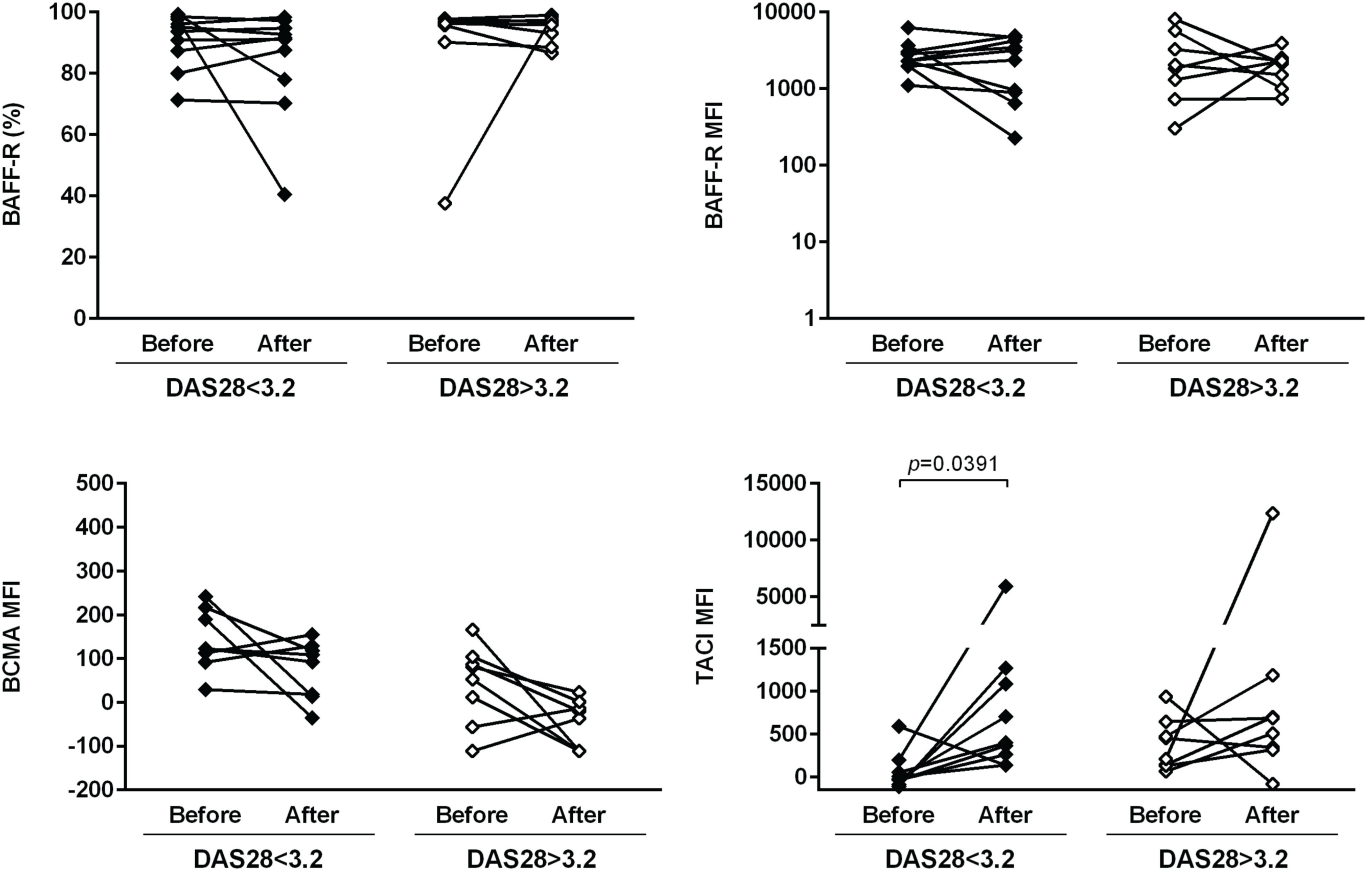
Rheumatoid arthritis patients with low to moderate disease activity after biologic therapy have increased TACI, but not BAFF-R or BCMA B-cell expression. The expression of B-cell activating factor receptors (BAFF-R, BCMA, TACI) was analyzed by flow cytometry (frequency and median fluorescence intensity, MFI) in established RA patients before and after an average of 8 months of biologic therapy with TNF-inhibitors and/ or tocilizumab, based on whether the patients presented a low to moderate disease activity (DAS28<3.2) or high disease activity (DAS28>3.2) after treatment. Differences were considered statistically significant for *p*<0.05 (Wilcoxon signed-rank test).

**Fig 13:**
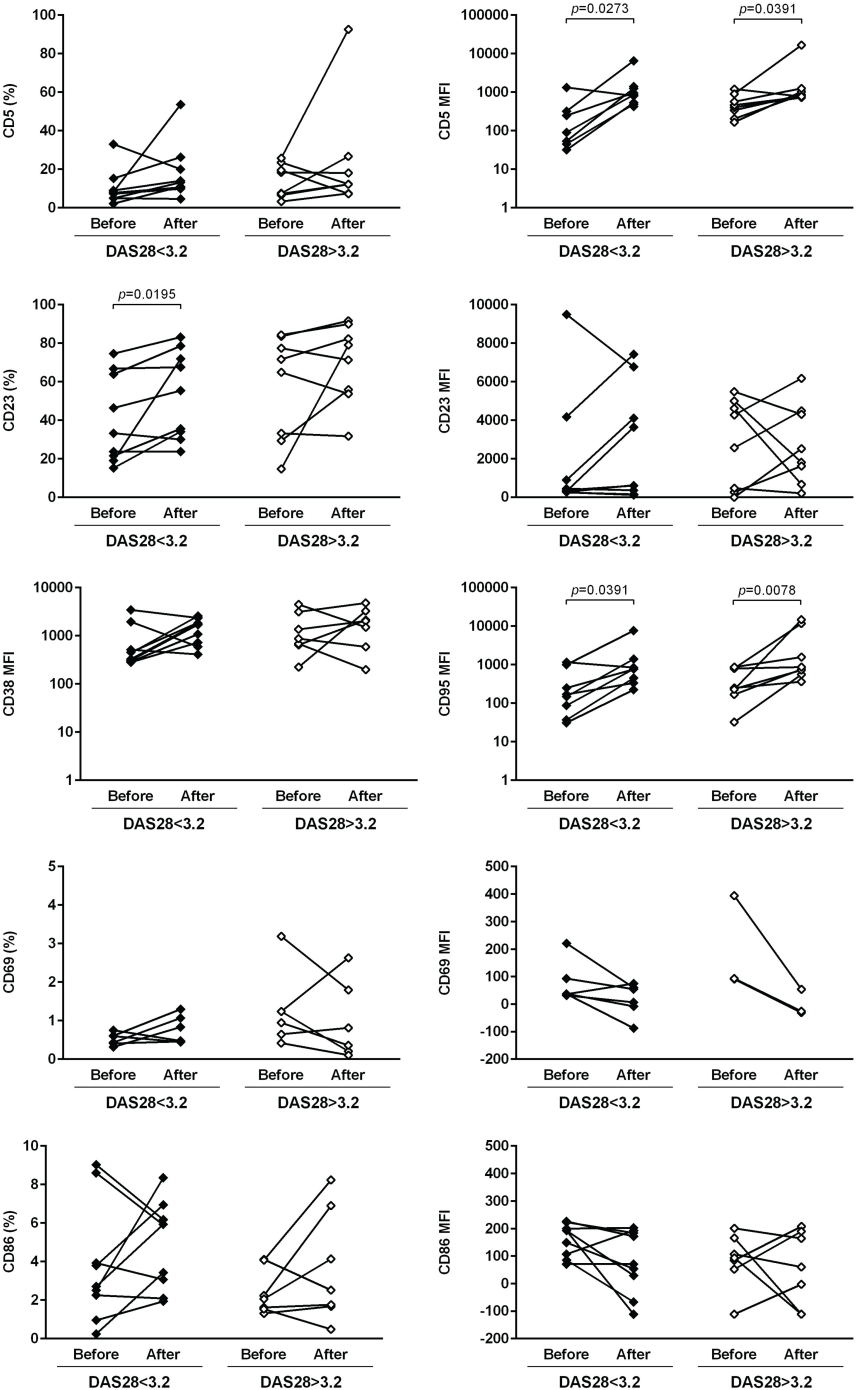
CD5 and CD95 B-cell expression increase in rheumatoid arthritis patients after biologic therapy irrespective of response to treatment. The expression of cell markers (CD5, CD23, CD38, CD69, CD86 and CD95) was analyzed on B-cells (frequency and median fluorescence intensity, MFI) by flow cytometry in established RA patients before and after an average of 8 months of biologic therapy with TNF-inhibitors and/ or tocilizumab, based on whether the patients presented a low to moderate disease activity (DAS28<3.2) or high disease activity (DAS28>3.2) after treatment. Differences were considered statistically significant for *p*<0.05 (Wilcoxon signed-rank test).

**Fig 14:**
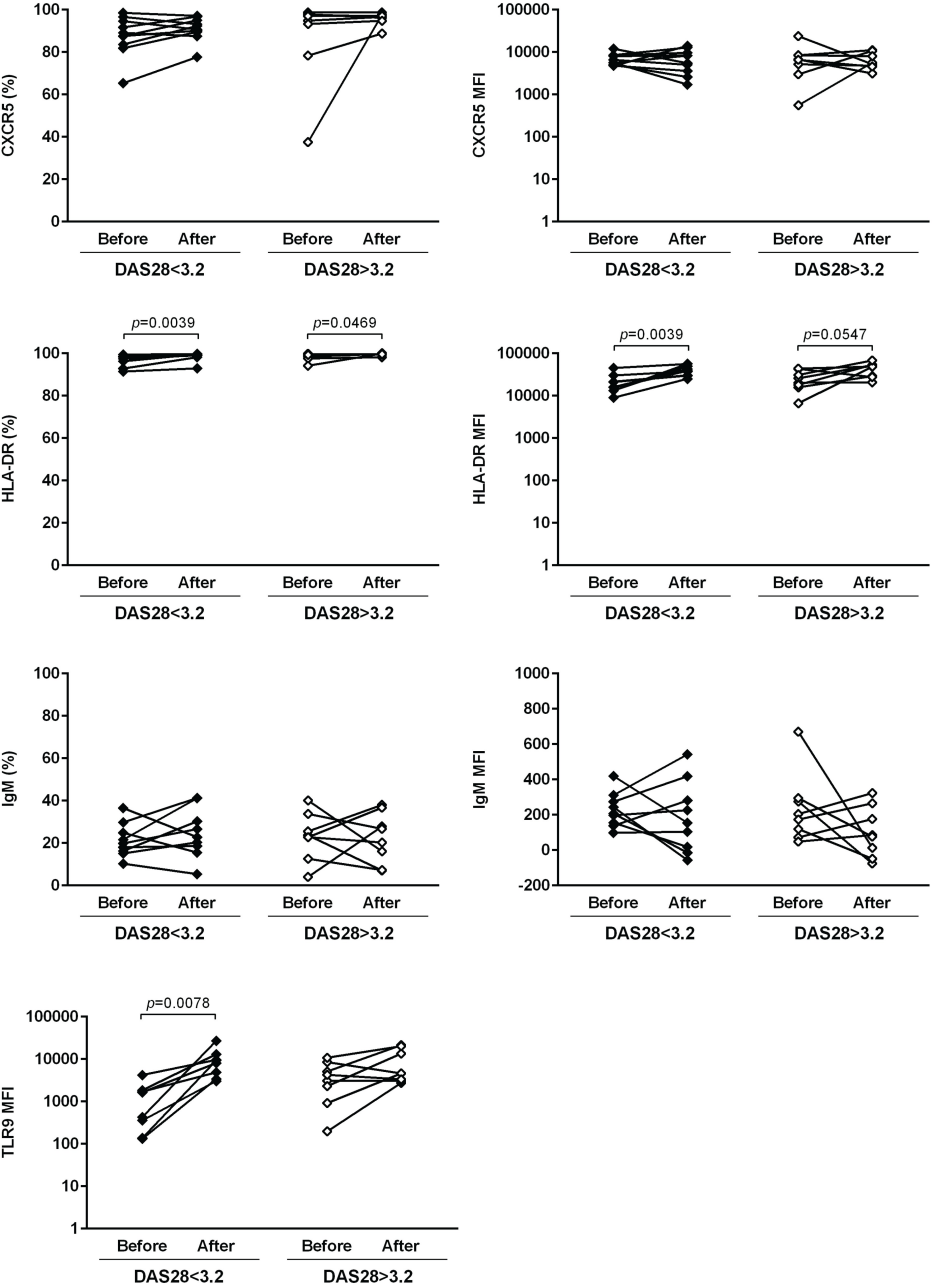
Biologic therapy affects not only the frequency of HLA-DR+ B cells in circulation, but also HLA-DR MFI in rheumatoid arthritis patients irrespective of response to treatment. The expression of cell markers (CXCR5, HLA-DR, IgM and TLR9) was analyzed on B-cells (frequency and median fluorescence intensity, MFI) by flow cytometry in established RA patients before and after an average of 8 months of biologic therapy with TNF-inhibitors and/ or tocilizumab, based on whether the patients presented a low to moderate disease activity (DAS28<3.2) or high disease activity (DAS28>3.2) after treatment. Differences were considered statistically significant for *p*<0.05 (Wilcoxon signed-rank test).

In order to understand if response to biologic therapy affected the results observed in circulating B cell subpopulations, B cell phenotype and B cell gene expression, we have analyzed RA patients before and after treatment with TNF-inhibitors and/ or tocilizumab based on whether the patients presented a low to moderate disease activity (DAS28<3.2) or high disease activity (DAS28>3.2) after treatment (Figs 11–14). Similarly to the results observed in Fig 1, we found that the frequency of total CD19+ B cells was not significantly affected after biologic therapy, irrespective of response to treatment. Nevertheless, the frequency of IgD-CD27- memory B cells was significantly decreased after biologic therapy not only in RA patients with low to moderate disease activity, but also in patients with high disease activity after treatment (Fig 11). Furthermore, no significant differences were observed in the frequencies of transitional, naïve B cells, pre-switch memory, post-switch memory and plasmablasts in RA patients after biologic therapy, irrespective of response to treatment (Fig 11). B cell phenotype analysis has shown that no significant differences were detected in BAFF-R (both frequency and MFI) and BCMA expression after biologic therapy irrespective of response to treatment, but TACI expression significantly increased in RA patients after biologic therapy who had a low to moderate, but not high, disease activity after treatment (Fig 12). No significant differences were observed in CD5+ B cells, but CD5 MFI values were significantly increased after biologic therapy, irrespective of response to treatment (Fig 13). The frequency of CD23+ B cells was significantly higher in RA patients after biologic therapy who had a low to moderate, but not high, disease activity after treatment, but no significant differences were observed in CD23 MFI values (Fig 13). CD38 MFI, CD69 and CD86 (both frequency and MFI) were not significantly different between RA patients after biologic therapy, irrespective of response to treatment (Fig 13). CD95 expression significantly increased in RA patients after biologic therapy not only in patients with low to moderate, but also high disease activity after treatment (Fig 13). HLA-DR expression (both frequency and MFI) significantly increased in RA patients after biologic therapy, irrespective of response to treatment (Fig 14), but no significant differences were observed in CXCR5 and IgM expression. TLR9 MFI was significantly increased in RA patients after biologic therapy who had a low to moderate, but not high disease activity after treatment (Fig 14). In addition, no significant differences were found in B cell gene expression and serum levels of CXCL13, sCD23 and BAFF in RA patients after biologic therapy, irrespective of response to treatment (data not shown).

## Discussion

In this study, the effect of TNF-inhibitors and tocilizumab treatments on peripheral blood B cell phenotype and B cell gene expression was analyzed in patients with RA. We found that untreated early RA and established RA patients have alterations in memory B cell subpopulations, particularly in DN (IgD-CD27-) memory B cells, and treatment with either TNF antagonists or tocilizumab restored the frequency of this B cell subset to normal levels. Furthermore, changes in the expression of some B cell markers such as TACI, TLR9, CD5, CD95 and HLA-DR, were also detected after treatment with TNF-inhibitors and/ or tocilizumab. No significant alterations were found in B cell gene expression, CXCL13, sCD23 and BAFF serum levels after treatment with TNF-inhibitors or tocilizumab.

Previous studies have demonstrated that alterations in B cell subpopulations, particularly memory B cell subsets, occur in RA patients, not only in peripheral blood, but also locally in the joints [3, 16, 17]. In fact, our group has described for the first time that untreated very early RA patients with less than 6 weeks of disease duration have significantly decreased circulating levels of pre-switch memory B cells (IgD+CD27+) [3], which can be related with B cell trafficking in the tissues and/ or with infiltration of the synovial membrane [16]. In this study, we found that untreated early RA patients (<1 year of disease duration, ERA) and established RA patients (MTX treated and before initiating anti-TNF or tocilizumab treatment) had significantly increased levels of DN (IgD-CD27-) B cells when compared to controls, which has also been recently described by others [18, 19]. Indeed, increased levels of IgD-CD27- memory B cells have been found in other autoimmune diseases such as systemic lupus erythematosus [20, 21] and multiple sclerosis [22]. Although the exact function of this B cell subset is not entirely understood, it is known that it belongs to the memory B cell compartment due to the high levels of somatic hypermutation [23–25]. Moreover, it has been suggested that these cells might contribute to inflammation by induction of T cell responses and the production of proinflammatory cytokines [22]. Also, since most of IgD-CD27- B cells were class-switched, it is possible that these B cells are linked with the production of higher affinity antibodies relevant in inflammation [26, 27]. Elevated numbers of DN memory B cells have also been related to aging [28, 29], but no correlation was found between the frequency of this B cell subpopulation and age in all studied groups, nor with inflammation parameters such as DAS28, ESR or CRP values. We found that treatment with TNF-inhibitors and tocilizumab restored the frequency of DN (IgD-CD27-) B cells to normal levels, which is in accordance with previous studies [16, 30–32], although there are some contradictory results [16, 33–35]. The disparities found between studies could be related to the use of different anti-TNF agents, disease activity, cohort size and previous treatments administrated to RA patients. Previous works have demonstrated that TNF-inhibitors affect memory B cell subpopulations not only in RA, but also in other inflammatory diseases [16, 19, 31, 36–39]. In fact, circulating naive B cells and class-switched memory B cells were found to be normally present in peripheral blood of patients with Crohn’s disease, whereas IgM+ memory B cell numbers were reduced. Nonetheless, a restoration of IgM+ memory B cell pool occured after treatment with infliximab [31, 36, 38]. Also, it has been shown that in patients with sarcoidosis, the increased blood levels of CD27-IgA+ memory B cells are normalized after treatment with infliximab [37]. Furthermore, it has been recently demonstrated that patients with Behçet's disease have significantly lower memory B cell numbers in peripheral blood, particularly CD27+IgA+ B cells, when compared to controls, but treatment with adalimumab restored to normal levels blood B cell numbers [39]. Of note, it has been previously shown that established RA patients have significantly lower levels of peripheral blood pre-switch IgD+CD27+ memory B cells when compared to healthy individuals, but treatment with infliximab restored the frequency of this B cell subpopulation to normal levels [16]. In the present study, the TNF-inhibitors analyzed were adalimumab (1 case), etanercept (5 cases) and golimumab (4 cases), which restored the frequency of IgD-CD27- memory B cells in RA patients to normal levels. Nevertheless, no significant differences were found in B cell subpopulations when TNF-inhibitors (etanercept and golimumab) were independently analyzed and compared (data not shown). Also, no significant differences were detected in B cell phenotype, B cell gene expression or serum levels of BAFF, CXCL13 and sCD23 before and after treatment with either golimumab or etanercept (data not shown). This is an important limitation of our study, since the low number of patients included of each anti-TNF treatment does not allow a robust statistical analysis of the individual TNF-inhibitors. Future studies with a higher number of RA patients included after treatment with TNF-inhibitors will be necessary to reinforce the conclusions regarding the effect of individual TNF-inhibitors on B cells. Tocilizumab has also been shown to induce a reduction in the frequency of memory B cell subpopulations in RA [40, 41], namely IgA+ and IgG+ B cells [40], which supports our results. Interestingly, it has been suggested that the frequency of DN (IgD-CD27-) B cells in RA might serve as a baseline predictor of subsequent response to tocilizumab treatment [18]. Possibly, the restoration of DN memory B cells to normal circulating levels in RA patients after treatment with either TNF-inhibitors or tocilizumab reflects a state of chronic B cell hyperactivity dependent on TNF and IL-6 [10, 11], which is inhibited by treatment. Of note, recent studies have demonstrated that microRNA-155, an important regulator of B cell activation, is highly expressed in peripheral blood B cells, particularly in IgD-CD27- memory B cells, in ACPA+ patients [27], which supports an enhanced activation of this particular B cell subset in RA. The normalization of IgD-CD27- B cells in peripheral blood after treatment with TNF-inhibitors and tocilizumab might suggest a relevant role of these B cells in RA pathogenesis either through T cell activation, cytokine release and/ or antibody production. Further studies are necessary in larger cohorts of patients to clarify the role of this B cell subpopulation in RA, particularly comparing seropositive and seronegative RA.

B cell phenotype analysis of ERA patients suggests an early B cell activation due to the increased frequencies of CD69+ B cells in circulation when compared to controls. Furthermore, the higher CD23 and CD38 MFI values observed in ERA patients reinforce an early B cell maturation and triggering, also supported by the increased serum levels of sCD23 detected in ERA [42, 43]. In addition, the higher CXCL13 (ligand for CXCR5 and a B cell chemotactic factor) serum levels observed not only in ERA, but also in established RA patients when compared to healthy individuals, support an active recruitment of B cells towards inflammatory sites since early RA onset [44][45]. Nevertheless, follicular T helper cells also express CXCR5 and respond to its ligand, CXCL13 [46]. Furthermore, it has been demonstrated that Th17 cells, known to contribute to RA pathogenesis, are an important source of CXCL13 and interact with B cells, triggering autoantibody production [47]. Therefore, the hypothesis that the increased serum levels of CXCL13 detected since early RA onset affect not only B cells, but also T cells, cannot be excluded. Thus, the recruitment and activation of CXCR5+ B and T cells, stimulated by CXCL13, towards joints and secondary lymphoid organs contribute to an exacerbation of the inflammatory process in RA [46, 48, 49]. Moreover, similarly to previous studies, alterations in the frequency of CD5+ B cells were detected in both ERA and established RA patients when compared to controls [50, 51]. CD5+ B cells are known to be associated with bone resorption through IL-6 production, a cytokine that supports osteoclast differentiation [52]. It is possible that the reduced circulating levels of CD5+ B cells are due to a recruitment of these cells towards the synovial tissue, where they might contribute to bone erosions.

Treatment with TNF-inhibitors and tocilizumab influenced some B cell surface markers. While no significant changes were observed in BAFF-R (both frequency and MFI values) or BCMA, TACI MFI increased after anti-TNF therapy. TACI plays an important role in humoral immunity [53], but its functional activity can be ambiguous as it can not only provide positive signals driving T-independent B cell responses and survival of activated B cells [54, 55], but also delivers negative signals suppressing B cell activation [56]. The increased TACI and CD95 MFI values detected after treatment with TNF-inhibitors might suggest an inhibition of B cell activation [56, 57]. Recent reports indicate that TNF-inhibitors modulate Fas-mediated apoptosis in RA [13, 58] and it has been demonstrated that anti-TNF treatment in RA inhibits B cell function by disrupting germinal centre reactions [14, 33] and decreasing T-cell dependent humoral responses [14]. B cell trafficking into inflamed tissues in RA is regulated by TNF and can be modulated not only by TNF-inhibitors [16, 59, 60], but also by tocilizumab treatment [61]. In this study, higher frequencies of HLA-DR+ B cells and HLA-DR MFI values were observed after treatment with both TNF-inhibitors and tocilizumab. It is possible that by inhibiting B cell recruitment and ameliorating cellular infiltration at inflammatory sites, treatment of RA patients with either TNF-inhibitors or tocilizumab leads to B cell recirculation through blood and lymphatic systems. These activated B cells have an altered phenotype, with changes in B cell activation markers such as HLA-DR, TLR9 and/ or chemokine receptors [14, 16, 30, 32, 57, 62], as observed in this study, which can be related with modifications on B cell triggering mechanisms potentiated by anti-TNF and/ or tocilizumab treatment. Furthermore, it was also found that the frequency of B cell subpopulations and B cell phenotype in established RA patients are affected by biologic therapy, irrespective of response to treatment. These results support the absence of correlation between circulating B cell levels, B cell phenotype and disease activity.

Our results also suggest that B cell gene expression in RA is not significantly affected by treatment with either TNF-inhibitors or tocilizumab, but alterations occur in ERA and established RA patients treated with MTX. The significantly increased BAFF-R, TACI and TLR9 B cell gene expression levels observed in established RA patients under MTX treatment when compared to ERA and controls support a relevant role of BAFF and T-cell independent mechanisms in B cell activation, particularly at later stages of RA development [4, 6, 63, 64]. Nevertheless, no significant differences were found at a protein level for BAFF-R and TACI MFI values, except for TLR9, whose expression was significantly increased in established RA when compared to controls. Thus, we cannot exclude the hypothesis that MTX treatment affects B cells at the gene, but not at the protein level, at least in the case of some cellular markers such as BAFF-R and TACI. Furthermore, BCL-2 gene expression levels were significantly increased in established RA, suggesting an inhibition of B cell apoptosis [65], which can be due to immunosuppressive treatment [66]. Similarly, the elevated FcγRIIB gene expression levels observed in established RA, when compared to ERA, might be related with suppression of B cell responses during MTX therapy [67]. Nonetheless, the higher β2M gene expression levels found in established RA support an increased B cell activation in chronic patients when compared to healthy individuals [68]. BLIMP-1, important for plasma cell differentiation [69], had B cell gene expression levels significantly increased in ERA patients in comparison with established RA under MTX treatment, which supports an activation of antibody secreting cells since early RA onset.

Overall, total CD19+ B cells were analyzed in this study and differences were only found in protein expression on B cells in some of the markers studied. Since alterations were detected in B cell subpopulations, particularly in IgD-CD27- memory B cells, after treatment with TNF-inhibitors and tocilizumab, future studies concerning an independent analysis of all B cell subsets (frequency and absolute numbers) would be fundamental. In fact, an analysis based on absolute numbers would be particularly relevant and more accurate to confirm any changes observed in circulating B cell subpopulations detected with relative numbers.

## Conclusions

To sum up, in RA, treatment with either TNF-inhibitors or tocilizumab affects B cell phenotype and the frequency of memory B cell subpopulations in peripheral blood, particularly DN (IgD-CD27-) B cells, but not B cell gene expression or serum levels of CXCL13, sCD23 and BAFF, when comparing baseline with post-treatment follow up. Overall, our results may suggest that TNF-inhibitors and tocilizumab inhibit B cell trafficking towards inflammatory sites, thus supporting activated B cell recirculation from tissues through blood and lymphatic systems.

## Supporting information

S1 Fig IgD-CD27- B cells are mainly class-switched and express low IgM levels. The frequency of IgD-CD27- B cells expressing IgM was determined by flow cytometry in early RA (ERA) and established RA patients under methotrexate treatment. In addition, the effect of TNF-inhibitors and tocilizumab treatment on IgM expression by IgD-CD27- B cells was also assessed in established RA patients at baseline and after an average of 8 months of treatment. A group of healthy individuals was also included as controls. Lines represent median values. Differences were considered statistically significant for *p*<0.05. Non-parametric Mann-Whitney test was used for comparisons between 2 independent groups. For paired samples (before and after treatment), the Wilcoxon signed-rank test was used.
(TIF)
