# Supplementary figures and images for: B-cell phenotype and IgD-CD27- memory B cells are affected by TNF-inhibitors and tocilizumab treatment in rheumatoid arthritis
Source: PLoS One. 2017 Sep 8;12(9):e0182927. doi: 10.1371/journal.pone.0182927 (PMC5590747; doi:10.1371/journal.pone.0182927)

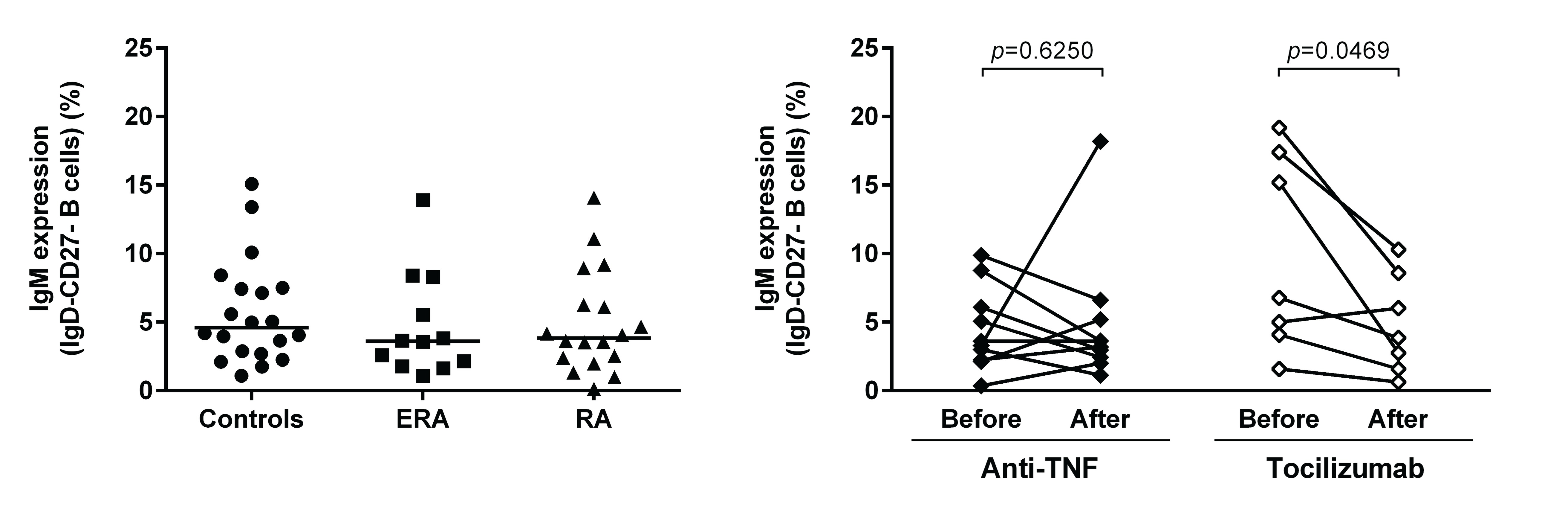

Supplement: S1 Fig — The frequency of IgD-CD27- B cells expressing IgM was determined by flow cytometry in early RA (ERA) and established RA patients under methotrexate treatment. In addition, the effect of TNF-inhibitors and tocilizumab treatment on IgM expression by IgD-CD27- B cells was also assessed in established RA patients at baseline and after an average of 8 months of treatment. A group of healthy individuals was also included as controls. Lines represent median values. Differences were considered statistically significant for p<0.05. Non-parametric Mann-Whitney test was used for comparisons between 2 independent groups. For paired samples (before and after treatment), the Wilcoxon signed-rank test was used. (TIF) [file pone.0182927.s001.tif]
